# Free Form Deformation–Based Image Registration Improves Accuracy of Traction Force Microscopy

**DOI:** 10.1371/journal.pone.0144184

**Published:** 2015-12-07

**Authors:** Alvaro Jorge-Peñas, Alicia Izquierdo-Alvarez, Rocio Aguilar-Cuenca, Miguel Vicente-Manzanares, José Manuel Garcia-Aznar, Hans Van Oosterwyck, Elena M. de-Juan-Pardo, Carlos Ortiz-de-Solorzano, Arrate Muñoz-Barrutia

**Affiliations:** 1 Biomechanics Section, Department of Mechanical Engineering, KU Leuven, 3001, Leuven, Belgium; 2 Instituto de Investigación Sanitaria-Hospital Universitario de la Princesa, Universidad Autonoma de Madrid, School of Medicine, 28006, Madrid, Spain; 3 Multiscale in Mechanical and Biological Engineering (M2BE), Department of Mechanical Engineering, Aragon Institute of Engineering Research (I3A), University of Zaragoza, 50018, Zaragoza, Spain; 4 Prometheus, Division of Skeletal Tissue Engineering, KU Leuven, Leuven, Belgium; 5 Regenerative Medicine Program, Institute of Health and Biomedical Innovation, Queensland University of Technology, 4059, Brisbane, Australia; 6 Cancer Imaging Laboratory, Program in Solid Tumors and Biomarkers, Center for Applied Medical Research (CIMA), University of Navarra, Navarra’s Health Research Institute (IDISNA), 31008, Pamplona, Spain; 7 Bioengineering and Aerospace Engineering Department, Universidad Carlos III de Madrid, Instituto de Investigación Sanitaria Gregorio Marañon, 28911, Madrid, Spain; University of Pittsburgh, UNITED STATES

## Abstract

Traction Force Microscopy (TFM) is a widespread method used to recover cellular tractions from the deformation that they cause in their surrounding substrate. Particle Image Velocimetry (PIV) is commonly used to quantify the substrate’s deformations, due to its simplicity and efficiency. However, PIV relies on a block-matching scheme that easily underestimates the deformations. This is especially relevant in the case of large, locally non-uniform deformations as those usually found in the vicinity of a cell’s adhesions to the substrate. To overcome these limitations, we formulate the calculation of the deformation of the substrate in TFM as a non-rigid image registration process that warps the image of the unstressed material to match the image of the stressed one. In particular, we propose to use a B-spline -based Free Form Deformation (FFD) algorithm that uses a connected deformable mesh to model a wide range of flexible deformations caused by cellular tractions. Our FFD approach is validated in 3D fields using synthetic (simulated) data as well as with experimental data obtained using isolated endothelial cells lying on a deformable, polyacrylamide substrate. Our results show that FFD outperforms PIV providing a deformation field that allows a better recovery of the magnitude and orientation of tractions. Together, these results demonstrate the added value of the FFD algorithm for improving the accuracy of traction recovery.

## Introduction

The quantification of cellular forces provides insights on the way that cells probe and respond to their microenvironment in a variety of physiological and pathological situations [1–4]. Traction Force Microscopy (TFM) [5] is a widespread method used to estimate cellular forces from the deformations that they cause on a flexible substrate that mimics the extracellular matrix (ECM) mechanical properties. Due to the optical clarity of the substrate, a large number of fluorescent beads are typically embedded into the hydrogel to serve as tracking points of its deformation, and image processing techniques are applied to compute the displacements of these beads. Since the Young’s modulus of the substrate is known, cellular forces can be retrieved from the previously computed displacement field assuming a known mechanical model of the substrate. TFM has been mainly used to this date for the quantification of 2D in-plane traction fields exerted by cells cultured on the surface of a planar substrate [5–7]. However, recent studies have revealed that cells exert forces in 3D and in this context, it is important to study the mechanical interactions of cells with their environment, which involve pushing as well as pulling forces in the out-of-plane direction [8–13].

**Fig 1:**
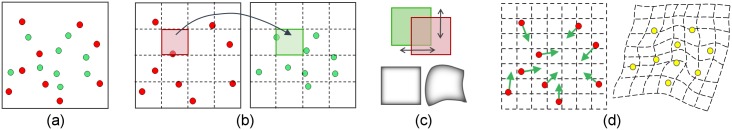
Schematic representation of the application of PIV and FFD algorithms to TFM. (a) Bead locations in the relaxed (red) and deformed (green) substrate. (b) In the block-matching (PIV) algorithm, the images of both relaxed (red) and deformed (green) substrates are divided into multiple blocks to perform a piece-wise comparison. (c) The deformation model for each block in PIV is usually assumed to be a rigid translation (top, in color); however, higher order non-rigid deformation models (bottom, in greyscale) have been also considered. (d) In the FFD algorithm, a uniform deformable mesh is used instead of blocks (left). The mesh is iteratively warped to align the beads of the relaxed substrate with their counterparts in the deformed substrate. At the end of the process (right), the beads have been registered (yellow) and the deformed mesh provides the displacement field required for the TFM analysis.

Cross-correlation based Particle Tracking Velocimetry (PTV) and Particle Image Velocimetry (PIV) are the preferred methods for tracking bead movements. PTV tracks the individual displacements of all the beads between the images of the deformed and non-deformed substrate [14]. This procedure can be computationally intensive and error-prone due to the large number of beads analyzed. In PIV, also known as block-matching or digital image correlation, the displacements are estimated from piecewise local correlations performed on image blocks [15] (Fig 1b). PIV assumes that the local deformation within each block can be approximated by simple rigid translations, which is often not the case. In addition, using PIV entails a loss in spatial resolution as it averages the displacements of the entire volume of the block. Several methods have been proposed to overcome these limitations by allowing non-rigid deformation of the blocks [16,17] (Fig 1c). To this end, each block deformation is fitted to a polynomial model whose parameters are iteratively computed. Once the parameters of the polynomial are estimated, the displacement field at any point within the block can be easily interpolated, thus avoiding the averaging effect. Unfortunately, this approach presents some drawbacks. First, the deformation order cannot be predicted beforehand and may vary in space. Thus, the degree of the fitting polynomial must be high enough to account for all the expected deformations. Since the number of parameters that need to be estimated increases with the polynomial degree, this escalates the computational complexity. Moreover, increasing the order of the polynomials tends to introduce artifacts such as ringing, ultimately producing unreliable results [18].

In this paper, we propose instead to use a B-spline-based Free Form Deformation (FFD) approach to estimate the displacements in TFM experiments. This technique provides a simple and powerful approach to model a wider range of local deformations overcoming most of the limitations presented by other existing methods. First, we show the potential of the proposed approach within a TFM framework. Next, we perform a systematic comparison of the quality of the traction reconstruction obtained from FFD-based displacements versus those obtained from the PIV algorithm. Finally, as proof of principle, we apply this technique to the 3D traction recovery of Human Umbilical Vein Endothelial Cells (HUVEC) lying on a soft substrate.

## Methods

### Free Form Deformation-Based Image Registration for Displacement Field Calculation

We formulate the calculation of the displacement field in TFM as the image registration problem of aligning the image of the deformed bead-containing substrate, *I*_*d*_, with the image of the relaxed substrate acquired after lysing the cell, *I*_*r*_. Image registration generally consists of three main elements [19–21]: a distance metric (e.g., cross-correlation, sum of squared differences, mutual information) to quantify the degree of alignment between the two images; a transformation model that defines a geometric mapping between the coordinates of both images; and an optimization strategy (e.g., gradient descendent, Gauss-Newton, Levenberg-Marquardt) that iteratively minimizes the cost function given by the distance metric in order to find the optimal parameters of the transformation model. Specifically, the distance metric is evaluated on each iteration, by comparing the deformed image and a warped version of the relaxed image. This warping process is a deformation applied to the relaxed image using the current estimate of the transformation model parameters. Once the distance metric has been evaluated, these parameters are updated as specified by the selected optimization strategy [20], and the process is repeated until the algorithm converges or a given ending criterion is reached.

The transformation function *T* is defined as the mapping of the coordinates **x** in the fixed image (*I*_*d*_) to the coordinates in the moving image (*I*_*r*_) by a certain displacement field **u**(**x**) that can be usually represented by a parametric model:

$$T_{p}\left(x\right)=x+u_{p}\left(x\right)$$ (1)

with **p** being the set of the parameters required for the selected transformation model. Hence, registering both images can be viewed as the problem of finding the displacement field that aligns *I*_*r*_ to *I*_*d*_:

$$\underset{u_{p}}{\text{min}}\text{ J}\left(u_{p};I_{r},I_{d}\left(T_{p}\right)\right)$$ (2)

with the cost function *J*(**u**_p_) given by:

$$\text{J}\left(u_{p}\right)=D\left(u_{p};I_{r},I_{d}\left(T_{p}\right)\right)+\gamma R\left(u_{p}\right)$$ (3)

where *D is the* distance (similarity) between both images and *γ* controls the amount of regularity *R* of **u**_**p.**_

In FFD-based image registration, the transformation model that warps the reference image during the optimization is given by a multivariate B-spline function whose control points are the tuning parameters [22,23]. The algorithm overlays a regular mesh over the fixed image *I*_*d*_ and defines the mesh nodes as the control points of B-splines curves. Then, the position of each of these control points is tuned iteratively during the optimization process deforming *I*_*r*_ until it matches *I*_*d*_. Fig 1d shows a schematic representation of the result of applying the FFD method to TFM. In 3D images, the *N*_*x*_×*N*_*y*_×*N*_*z*_ mesh of control points consists of a discrete set of uniformly spaced points *ϕ*_*i*,*j*,*k*_ with −1 ≤ *i* ≤ *N*_*x*_−1, −1 ≤ *j* ≤ *N*_*y*_−1 and −1 ≤ *k* ≤ *N*_*z*_−1. The constant spacing between the control points in the X, Y and Z-Cartesian direction is denoted by Δ_*x*_, Δ_*y*_ and Δ_*z*_, respectively. Then, in the case of using cubic B-splines as warping functions, the transformation that represents the local deformations and maps the voxel coordinates **x** = (*x*,*y*,*z*) of *I*_*d*_ to the voxel coordinates of *I*_*r*_ is:

$$T_{\Phi }\left(x\right)=x+\Sigma_{r=0}^{3}\Sigma_{s=0}^{3}\Sigma_{t=0}^{3}B_{r}\left(q\right)B_{s}\left(v\right)B_{t}\left(w\right)\phi_{i+r,j+s,k+t}$$ (4)

Here, *i* = ⌊*x*/Δ_*x*_⌋−1, *j* = ⌊*y*/Δ_*y*_⌋−1 and *k* = ⌊*z*/Δ_*z*_⌋−1 denote the index of the control point cell containing (*x*,*y*,*z*), *q* = (*x*/Δ_*x*_)−⌊*x*/Δ_*x*_⌋, *v* = (*y*/Δ_*y*_)−⌊*y*/Δ_*y*_⌋ and *w* = (*z*/Δ_*z*_)−⌊*z*/Δ_*z*_⌋ are the relative positions of (*x*,*y*,*z*) inside that cell in three dimensions, and the functions *B*_*r*_, *B*_*s*_ and *B*_*t*_ are the cubic B-spline polynomials [22] defined as follows:

$$\begin{array}{l} B_{0}\left(q\right)={\left(1-q\right)}^{3}/6\\ B_{1}\left(q\right)=\left(3q^{3}-6q^{2}+4\right)/6\\ \begin{array}{l} B_{2}\left(q\right)=\left(-3q^{3}+3q^{2}+3q+1\right)/6\\ B_{3}\left(q\right)=q^{3}/6 \end{array} \end{array}$$ (5)

Note that, once the registration is completed, the displacement field for each voxel can be easily obtained from the final configuration of the control points and the used B-spline model.

Regarding the distance metric and the optimization strategy, most of the methods available in the image registration literature [20] may be applied. In this study we used the normalized correlation coefficient as the distance metric. Then, since we do not use regularization (i.e. *γ* = 0), the cost function given in Eq 3 can be rewritten as

$$\text{J}\left(u_{p}\right)=D\left(u_{\Phi };I_{r},I_{d}\left(T_{\Phi }\right)\right)=\frac{\sum_{x}\left(I_{d}\left(x\right)-\bar{I_{d}}\right)\left(I_{r}\left(T_{\Phi }\left(x\right)\right)-\bar{I_{r}}\right)}{\sqrt{\sum_{x}{\left(I_{d}\left(x\right)-\bar{I_{d}}\right)}^{2}\cdot \sum_{x}{\left(I_{r}\left(T_{\Phi }\left(x\right)\right)-\bar{I_{r}}\right)}^{2}}}$$ (6)

with $\bar{I_{d}}=\sum_{x}I_{d}\left(x\right)$ and $\bar{I_{r}}=\sum_{x}I_{r}\left(x\right)$ being the average grey-values of the images being registered. Finally, a stochastic gradient descent method with adaptive estimation of the step size has been selected as the optimizer [24]. This optimization strategy relies on the random sampling of the data in the computation of the gradient at each iteration, requiring little computation time per iteration. Moreover, it works with an adaptive size prediction, which improves the robustness with respect to the particular parameters set by the user.

Several remarks have to be made. First, since the control points of the B-spline curves are moved independently, the registration causes a non-rigid deformation of the space between them. Furthermore, the movement of each control point is driven only by the displacements of the surrounding beads. Therefore, there is no need for prior knowledge or assumptions about the order of the underlying local deformation. Indeed, FFD provides a large number of degrees of freedom to cope with large deformations through the number of control points in the mesh. Finally, B-splines have a compact support, which means that the transformation of any image coordinate can be computed from a few surrounding control points (4x4x4 neighboring control points for 3D images using cubic B-splines). This is beneficial both for modeling local transformations, and for fast computation.

### Fourier Domain Traction Recovery

We analyzed the tractions exerted by cells lying on the surface of a thick substrate. Under this assumption, the substrate can be considered a semi-infinite linear half space. The 3D cell tractions were recovered by Tikhonov regularized inversion of the elasticity problem in the Fourier domain. To this end, we first extended to 3D the Fourier Transform Traction Cytometry algorithm proposed by Butler and co-workers [5]. Specifically, an integral Boussinesq analytical equation [25,26] was used as Green function to avoid the singularity that the original solution presents at the origin, and the tractions were recovered by solving the following least squares minimization problem in the Fourier domain:

$$\underset{\tilde{T}}{\text{min}}\left\{\tilde{∥u}-\tilde{G}\tilde{T}{∥}^{2}+\lambda ∥\tilde{T}{∥}^{2}\right\}$$ (7)

Here, $\tilde{G}$, $\tilde{T}$ and $\tilde{u}$ represent the three-dimensional integral Boussinesq Green’s function expressed as a tensor, the traction field and the displacement field, respectively, in the Fourier domain and *λ* is a parameter that controls the amount of regularization applied, which has been automatically selected based on the L-curve criterion.

### Implementation

Except for the displacement field estimation, the TFM computational workflow has been built using custom-made software programmed in Matlab (The Mathworks Inc., Natick, MA USA). Due to its versatility and efficiency, FFD-based displacement field estimation was computed using elastix [27], an open-source multiplatform software for image registration. The integration of the elastix-based FFD image registration on the TFM workflow has been done through a custom-made Matlab wrapper function. In particular, Matlab launches elastix providing it with the bead images and the necessary user parameters (the mesh size, the optimizer, the number of random samples per iteration and the number of iterations). Once the registration is complete, the wrapper reads and reshapes the obtained displacement field into an appropriate format and scales it with the user defined voxel size (usually given in microns). Note that we have restricted the number of parameters that can be tuned during the registration with elastix to those that directly depend on the TFM experimental setup.

A software package implementing this approach is provided as S2 File.

## Evaluation on Synthetic Data

### Evaluation Conditions

Legant et al. [9] suggested that TFM algorithms should be characterized by a combined analysis of their spatial resolution and traction sensitivity, which are inherently interdependent and account for the minimum detectable magnitude of tractions exerted within a given area. This can be easily done *in silico* using computer simulations of uniform tractions aligned with the X, Y, or Z Cartesian directions and distributed over a circular area. Here, we have made use of a previously developed TFM model [26] that simulates the 3D displacement field produced by different traction patterns exerted on the surface of an elastic substrate.

To assess the performance of the proposed algorithm, we used our TFM simulator to generate 3D images of relaxed and stressed substrate volumes containing embedded fluorescent beads as acquired by an optical microscope, for different combinations of applied traction area, magnitude and direction. These images were then fed to the algorithms under evaluation and the recovered displacement and force fields were compared to the ground-truth given by the simulated fields. Note that the same performance would be expected for tractions aligned with the X and Y Cartesian directions under the linear half space approximation and, thus, only one of them needs to be evaluated.

### Error Metrics

TFM is an ill-posed inverse problem where the displacement field contains errors of different nature. Therefore, it is difficult to define an independent error metric for the displacements that correlates with the accuracy of their corresponding tractions. Instead, we have used the quality of the retrieved tractions to compare the performance of the displacement estimation algorithms.

We have evaluated the error in the recovery of the traction field within a region of interest defined by the stress footprint. This footprint has been automatically obtained by segmenting the magnitude of the recovered tractions using a fixed threshold equal to 50% of its maximum value. Note that there is a single circular traction area per simulated traction field. Therefore, the recovery of multiple stress footprints would indicate failure to solve the inverse problem, making the algorithm under evaluation unsuitable for that given condition and discarding it for further analysis. For those evaluation conditions where the segmentation provides a single stress footprint, the quality of the resulting tractions has been determined using four metrics: the errors in the magnitude and direction of the recovered traction field and in the size and shape of the segmented stress footprint. Being ***T*** the retrieved traction field, ***T***_*gt*_ the simulated ground-truth traction field, and *N* and *M* the total number of points within the retrieved and ground-truth stress footprints, respectively, the error metrics are defined as follows:

*Magnitude error*. Absolute error (in percentage) of the total force magnitude within the stress footprint:

$$e_{\text{mag}}=100\cdot \left|\frac{\sum_{n=1}^{N}∥T{∥}_{n}\cdot \Delta_{xy}^{2}-\sum_{m=1}^{M}∥T_{gt}{∥}_{m}\cdot \Delta_{xy}^{2}}{\sum_{m=1}^{M}∥T_{gt}{∥}_{m}\cdot \Delta_{xy}^{2}}\right|$$ (8)

where Δ_*xy*_ is spatial resolution (pixel size) of the traction field.

*Direction error*. Average angular error within the segmented stress footprint, weighted at each point by the recovered traction magnitude:

$$e_{\text{ang}}=\frac{1}{N}\Sigma_{n=1}^{N}\left(\frac{∥T{∥}_{n}}{\sum_{n=1}^{N}∥T{∥}_{n}}\right){\text{ cos}}^{-1}\left(\frac{T_{n}\cdot T_{gt}}{∥T{∥}_{n}∥T{∥}_{gt}}\right)$$ (9)

*Stress footprint size error*. Absolute percentage error of the segmented stress footprint area:

$$e_{\text{size}}=100\cdot \left|\frac{A-A_{gt}}{A_{gt}}\right|$$ (10)

with *A* and *A*_*gt*_ being the area of the recovered and ground-truth footprints, respectively.

*Stress footprint shape error*. Circularity of the segmented area:

$$c=\frac{4\pi A}{P^{2}}$$ (11)

where *P* is the perimeter of the segmented footprint and *c*∈[0,1], with *c* = 1 only if the shape of the segmented area is a circle.

## Results

### Performance of FFD for Displacement Calculation in TFM Experiments

**Fig 2:**
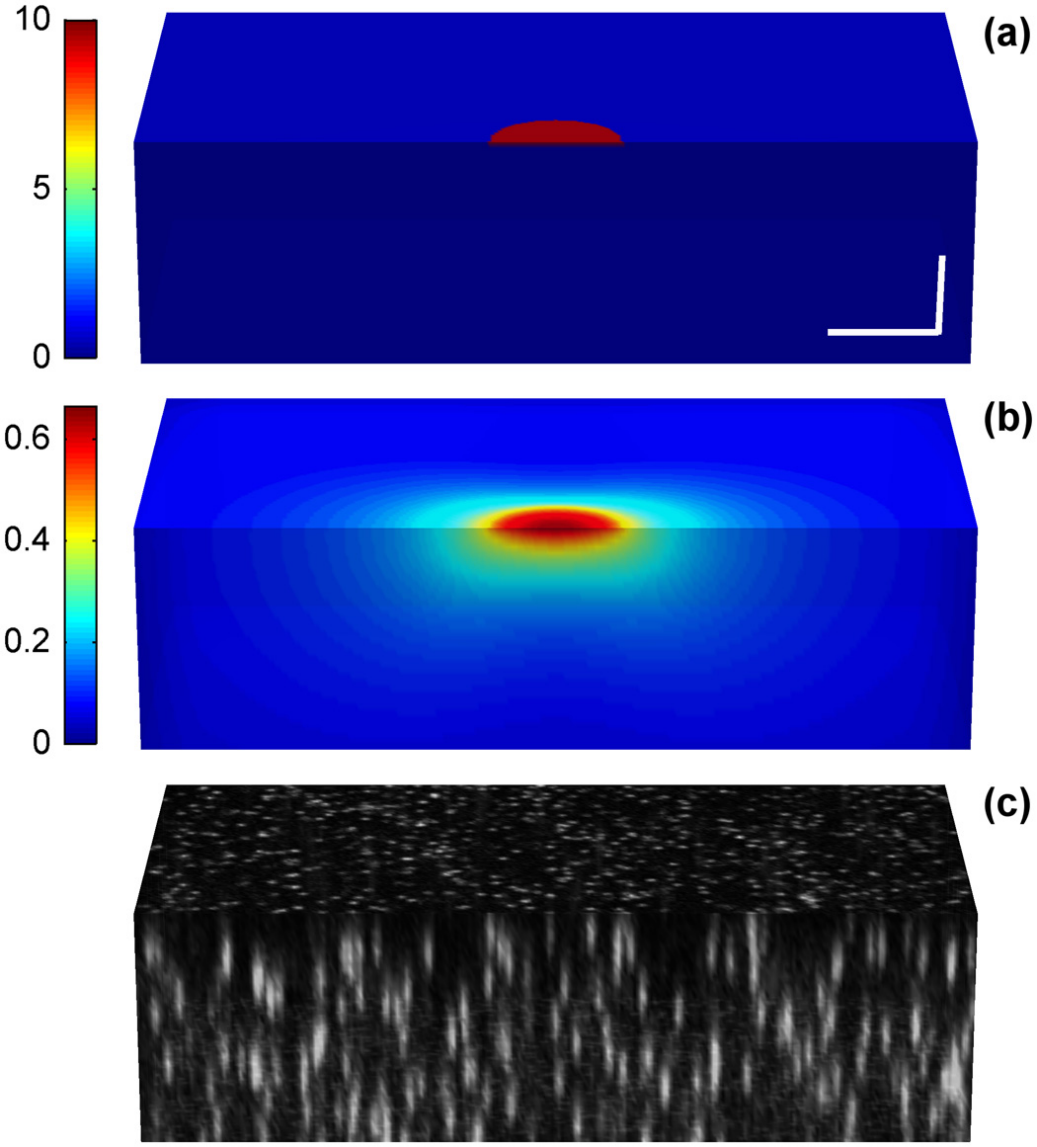
Example of a simulated TFM data set for a circular traction patch of 6μm diameter exerting a load of 10% of the Young’s modulus along the X-axis. (a) Magnitude of the traction field, (b) magnitude of the displacements caused by the tractions in (a), and a simulated substrate volume containing 0.2μm fluorescent beads (c). Units of the tractions are given as percentage of the Young modulus. Units of the displacements are given in μm. The scale bars represent 5μm.

We used our TFM simulator [26] to generate 18 artificial experimental set-ups consisting of uniform tractions with a magnitude of 15%, 10% or 5% of the substrate Young’s modulus, aligned with the X or Z Cartesian directions, and distributed over a circular area of 10μm, 6μm or 4μm diameter located on the surface of a linear elastic half space. For each test condition, we generated images of two 90μm thick substrates, one under stress and one relaxed. The parameters of the simulated substrate and the imaging system were selected to mimic realistic experimental situations. Specifically, the images of the substrate were generated as acquired by a laser scanning confocal microscope with a 60X (1.30 NA) objective lens providing a final voxel size of 0.15μm in the XY-plane and 0.3μm along the Z-axis. The Young’s modulus, the Poisson ratio, and the refractive index of the substrate were set to 5kPa, 0.45 and 1.39, respectively. The substrates contained 0.2μm fluorescent beads (λ_em_ = 605nm) at a density of 1bead/μm^3^. Note that the embedded beads were randomly distributed and, thus, multiple realizations were needed to provide reliable performance results. Hence, we have simulated and analyzed 20 realizations of the substrate volume images for every condition. Fig 2 and S1 Fig show a summary of this process for a circular traction patch of 6μm diameter exerting tractions with magnitude of 10% of the Young’s modulus and oriented along the X- and Z- Cartesian directions, respectively.

Next, we fed the simulated substrate images to the TFM software, and the quality of resulting 3D tractions was used to evaluate the performance of the displacement estimation algorithm. In this study, we have compared the performance of the FFD algorithm to the classic rigid block-matching approach, which has been implemented by extending to the third dimension the PIV scheme typically used in TFM experiments [15] and replacing the correlation-based block shift calculation by the more accurate iterative gradient-based optimal estimator [28]. As previously explained, the FFD–based image registration was implemented using elastix and integrated into the general TFM workflow. We used normalized correlation as distance metric and the adaptive stochastic gradient descent optimization. The maximum number of iterations of the optimizer was set to 2000 and the number of random spatial samples per iteration was set to 5000. The remaining parameters and settings were either automatically estimated from the data by elastix or kept at their default values (see S2 File). Both FFD and PIV were implemented in a three-level multi-scale strategy. Note that, for the finest scale, the grid size of control points (the block size) in FFD (PIV) depends not only on the bead density but also on the magnitude of the exerted tractions and the area over which they are distributed. Since a single cell usually exerts tractions of different magnitudes over focal adhesions of diverse sizes, we cannot fit a grid/block size independently for each condition. Instead, we used an average grid/block size value appropriate for most of the evaluated conditions. This value was 2.25x2.25x4.5μm for FFD and 2.7x2.7x5.4μm for PIV. Finally, the displacements calculated by PIV were interpolated to the original volume size to allow for a comparison with the FFD outcomes and simulated ground-truth data.

**Fig 3:**
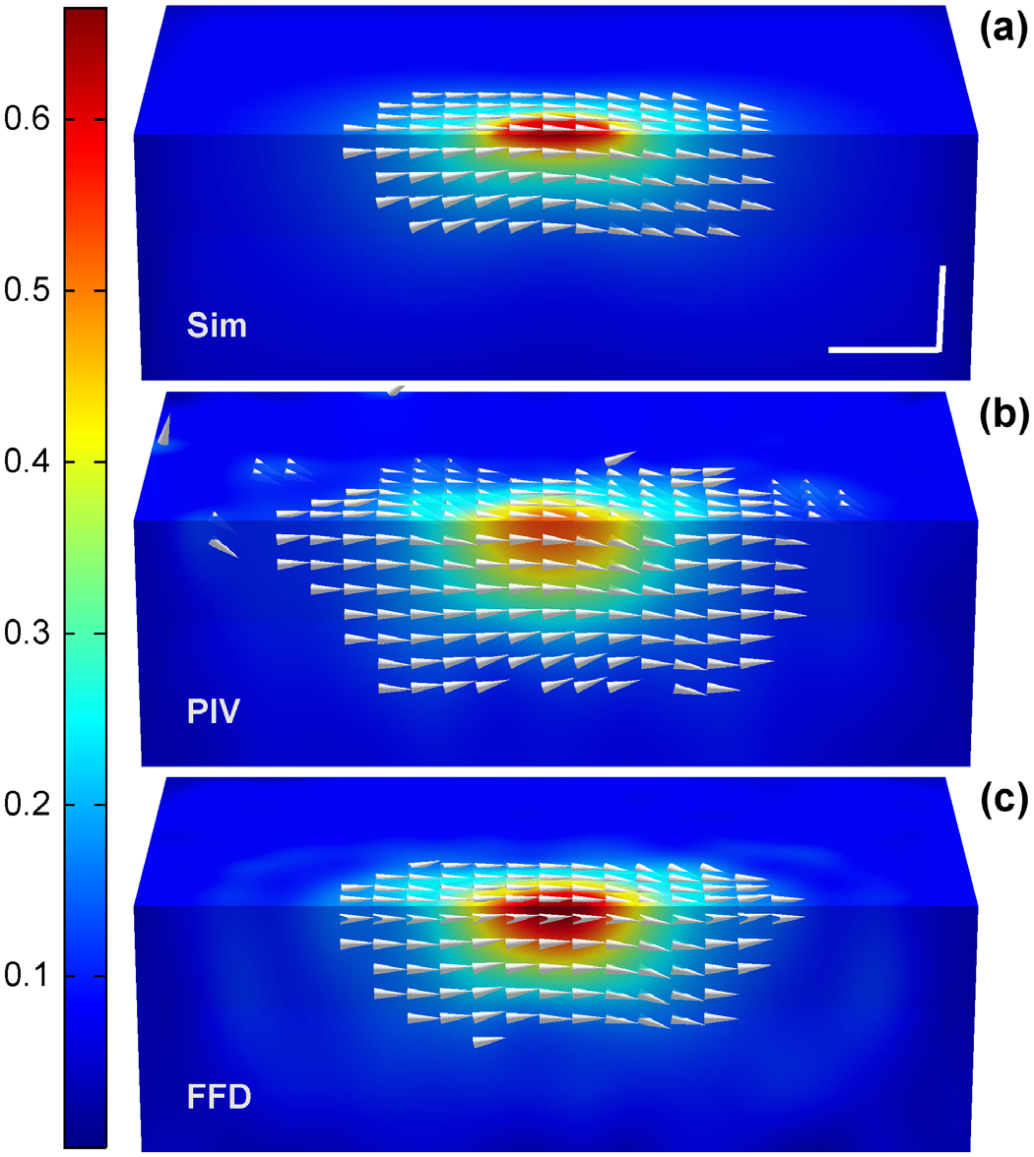
Displacements obtained from a circular traction patch of 6μm diameter exerting a load of 10% of the Young modulus along the X-axis. (a) Magnitude of the displacement field provided by the simulated ground-truth data, (b) PIV algorithm and (c) FFD algorithm. Cones indicate the direction of the field at those locations where the magnitude is larger than 20% of the peak magnitude. Units are given in μm. The scale bars represent 5μm.

**Fig 4:**
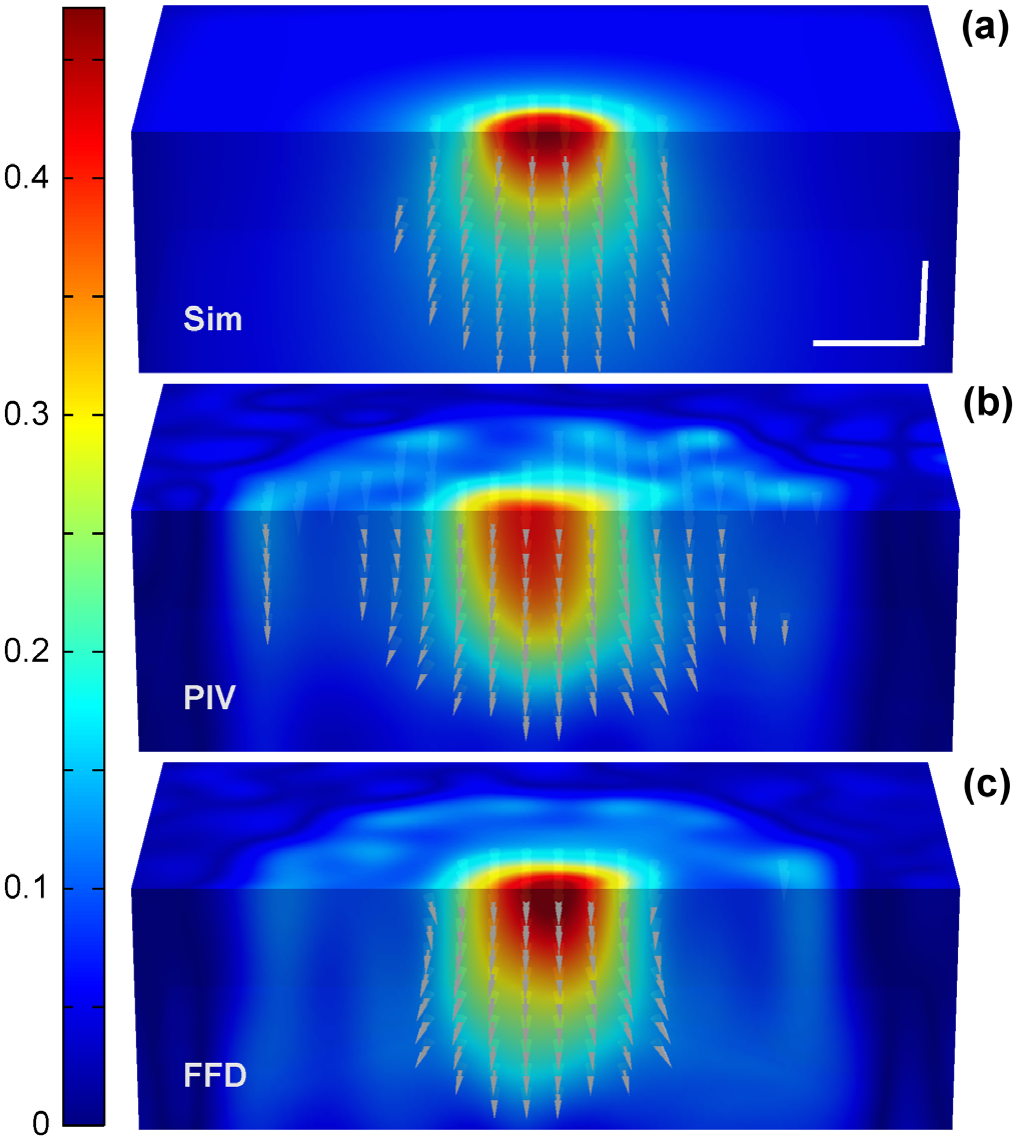
Displacements obtained from a circular traction patch of 6μm diameter exerting a load of 10% of the Young modulus along the Z-axis. (a) Magnitude of the displacement field provided by the simulated ground-truth data, (b) PIV algorithm and (c) FFD algorithm. Cones indicate the direction of the field at those locations where the magnitude is larger than 20% of the peak magnitude. Units are given in μm. The scale bars represent 5μm.

Figs 3 and 4 show a representative example of the displacements obtained by FFD and PIV when tractions were exerted along the X and Z Cartesian axis, respectively. Note that since the Green function has–by definition- an infinite support, the displacements are always distributed over the whole volume. However, a fast decay of the magnitude makes them visually negligible at a certain distance from the area where the traction is exerted. In Figs 3 and 4, the cones indicating the direction of the displacement field are only visible at those locations where the magnitude is larger than the 20% of the peak magnitude. Therefore, Figs 3 and 4 qualitatively show that FFD provides a more reliable displacement field, not only in magnitude but also in distribution (i.e., in their decaying rate). In contrast, PIV presents displacements of lower magnitude and decaying rate, causing relative larger errors in locations where the magnitude of the ground-truth displacements are almost negligible.

**Fig 5:**
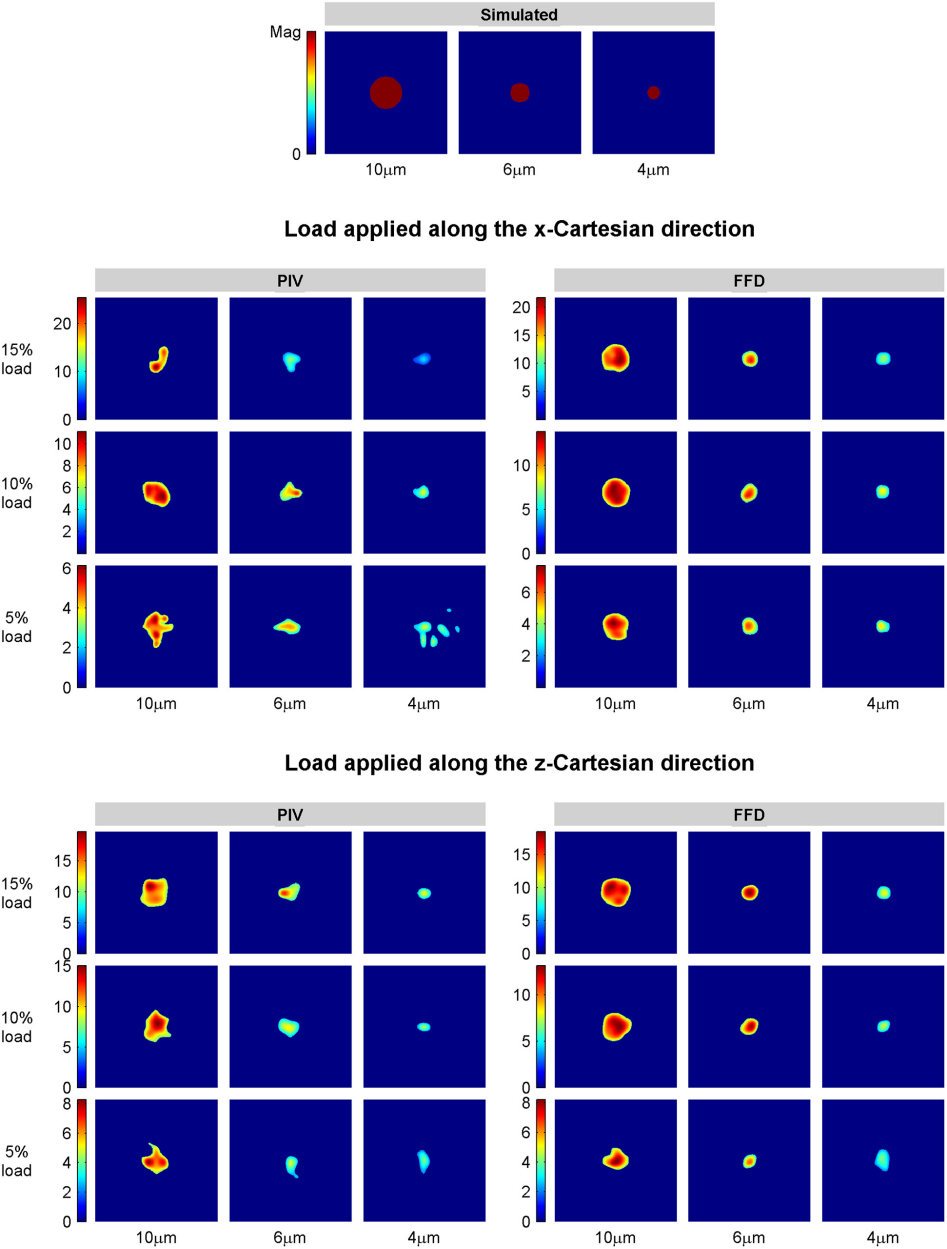
Recovered stress footprints. Recovered stress footprints for tractions with magnitudes of 15%, 10% and 5% of the substrate Young’s modulus, aligned with the X and Z Cartesian directions, and distributed over a circular area of 10μm, 6μm and 4μm diameter. Units of color bars are given as percentage of the Young’s modulus.

To quantify the performance of the TFM algorithms, we first segmented the stress footprint from the magnitude of the recovered tractions (see Fig 5). If more than the 20% (4 out of 20) of the realizations for a given test condition showed more than one segmented area, we concluded that the algorithm used to calculate the displacements was not suitable for that condition and we did not carry the analysis further. S1 Table shows the percentage of realizations that rendered a single stress footprint. As shown, traction retrieval is achieved by FFD in all the evaluated conditions while PIV fails for small patches (4μm size) that exert weak tractions (magnitude of 5%) along the X-axis. In those cases a single stress footprint is obtained in only 45% of the realizations.

#### Recovered total force magnitude and average direction

We then calculated the error in the total force magnitude and the average orientation retrieved within the segmented area in those conditions that resulted in a single stress footprint. Since the calculation of the displacements is affected by the bead image quality, different performance results should be expected depending on the orientation of the tractions as the point spread function of the microscope shows different spreading along XY-plane and Z-direction.

**Fig 6:**
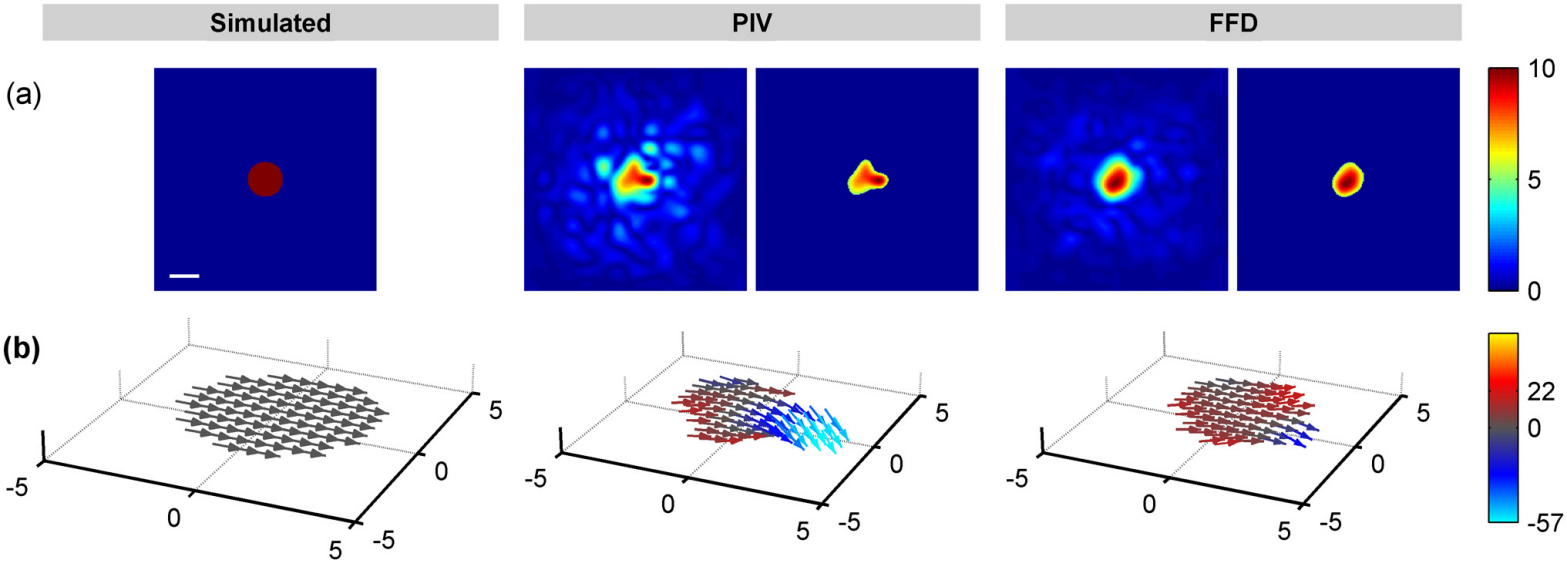
Tractions obtained from a circular traction patch of 6μm diameter exerting a load of 10% of the Young’s modulus along the X-axis. From left to right, simulated tractions, results from PIV, and results from FFD. (a) Magnitude of the traction field before and after segmenting the stress footprint and, (b) its corresponding orientation with the elevation angle indicated by the colormap. Units of the magnitude are given as percentage of the Young’s modulus. Units of the elevation angles (with respect to X-axis) are given in degrees. The scale bar represents 5μm.

**Fig 7:**
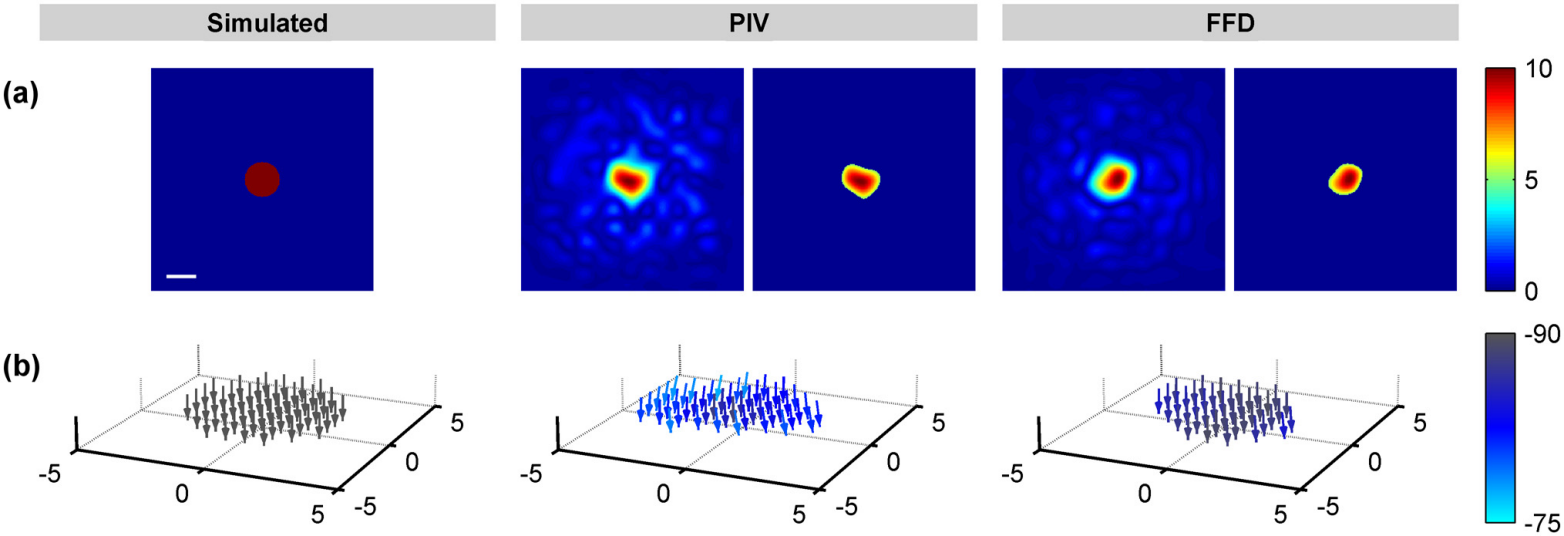
Tractions obtained from a circular traction patch of 6μm diameter exerting a load of 10% of the Young modulus along the (negative) Z-axis. From left to right, simulated tractions, results from PIV, and results from FFD. (a) Magnitude of the traction field before and after segmenting the stress footprint and, (b) its corresponding orientation with the elevation angle indicated by the colormap. Units of the magnitude are given as percentage of the Young modulus. Units of the elevation angles are given in degrees (-90 corresponding to negative Z-axis). The scale bar represents 5μm.

Figs 6 and 7 show the recovered traction magnitude and orientation for a 6μm size patch exerting tractions equal to 10% of the Young’s modulus along the X and Z-axis, respectively. Additionally, Fig 5 displays representative examples of every condition for magnitude and S2 and S3 Figs for orientation. These data indicate that, whereas both FFD and PIV show similar performance with applied out-of-plane tractions, FFD leads to the recovery of smoother magnitudes and improved directions for applied in-plane traction fields.

Our qualitative observations support the numerical results (see Table 1). Tractions retrieved from FFD-based displacements had smaller errors in both magnitude and angle for all tested conditions when in-plane tractions were applied. Specifically, FFD returned magnitude average errors 10%–40% smaller than those returned by PIV, with a maximum reduction of more than 50%. Regarding the orientation, FFD keeps the error below 10° for most of the conditions while PIV shows angular errors of 20° on average. Furthermore, the large variability in the performance of the PIV algorithm for different realizations suggests that PIV is more sensitive than the FFD to the specific distribution of the fluorescent beads.

**Table 1:** Quantitative performance evaluation of the recovered total force magnitude and average orientation within the segmented stress footprint for FFD and PIV algorithms within a TFM framework.

		FFD									PIV								
		10um			6um			4um			10um			6um			4um		
		15%	10%	5%	15%	10%	5%	15%	10%	5%	15%	10%	5%	15%	10%	5%	15%	10%	5%
**Load along X-axis**	**Mag. Error (%)**	*15*.*49 ± 2*.*57*	*15*.*82 ± 3*.*68*	*17*.*13 ± 3*.*33*	*29*.*69 ± 3*.*58*	*26*.*46 ± 2*.*96*	*28*.*45 ± 6*.*64*	*25*.*6 ± 3*.*88*	*23*.*72 ± 6*.*19*	*19*.*35 ± 6*.*44*	*59*.*04 ± 20*.*18*	*40*.*77 ± 7*.*53*	*35*.*9 ± 6*.*54*	*49*.*41 ± 10*.*58*	*43*.*6 ± 11*.*34*	*40*.*76 ± 12*.*06*	*51*.*98 ± 5*.*78*	*47*.*64 ± 11*.*77*	*-*
	**Ang. Error (°)**	*5*.*99 ± 0*.*89*	*6*.*61 ± 1*.*14*	*9*.*35 ± 1*.*29*	*6*.*46 ± 1*.*53*	*7*.*83 ± 2*.*51*	*9*.*76 ± 2*.*34*	*9*.*15 ± 2*.*68*	*9*.*43 ± 3*.*23*	*11*.*44 ± 2*.*45*	*26*.*94 ± 19*.*7*	*17*.*3 ± 3*.*6*	*23*.*38 ± 4*.*89*	*18*.*49 ± 5*.*35*	*20*.*83 ± 4*.*88*	*25*.*98 ± 6*.*9*	*16*.*62 ± 6*.*15*	*20*.*56 ± 6*.*26*	*-*
**Load along Z-axis**	**Mag. Error (%)**	*21*.*9 ± 2*.*91*	*19*.*94 ± 4*.*93*	*43*.*69 ± 10*.*44*	*34*.*49 ± 2*.*52*	*25*.*25 ± 5*.*06*	*56*.*83 ± 5*.*26*	*33*.*73 ± 5*.*32*	*48*.*95 ± 8*.*2*	*11*.*25 ± 7*.*6*	*24*.*79 ± 4*.*54*	*23*.*24 ± 9*.*9*	*36*.*46 ± 9*.*32*	*33*.*25 ± 8*.*41*	*30*.*08 ± 8*.*9*	*56*.*08 ± 6*.*25*	*46*.*31 ± 6*.*7*	*58*.*38 ± 7*.*07*	*13*.*45 ± 10*.*88*
	**Ang. Error (°)**	*3*.*09 ± 0*.*47*	*3*.*56 ± 0*.*57*	*5*.*28 ± 0*.*81*	*6*.*38 ± 1*.*42*	*2*.*83 ± 0*.*62*	*3*.*82 ± 0*.*71*	*7*.*56 ± 1*.*73*	*8*.*16 ± 1*.*52*	*4*.*59 ± 1*.*2*	*2*.*69 ± 0*.*41*	*3*.*1 ± 0*.*62*	*6*.*36 ± 0*.*81*	*4*.*1 ± 0*.*72*	*5*.*4 ± 1*.*17*	*3*.*8 ± 0*.*8*	*3*.*75 ± 0*.*38*	*3*.*66 ± 1*.*06*	*3*.*83 ± 0*.*9*

Evaluation conditions include 20 realizations with loads with magnitudes of 15%, 10% and 5% of the substrate Young’s modulus, aligned with the X and Z Cartesian direction and distributed over a circular area of 10μm, 6μm and 4μm diameter. Magnitude errors are given in percentage and angular errors in degrees.

To better understand why PIV performs worse than FFD for the recovery of in-plane tractions, we did a more in depth analysis of each vector component for the calculated displacement and traction fields. S4–S6 Figs show that PIV estimates the displacement fields in the XY-plane reasonably well: distributions agree with the ground truth, although magnitudes are underestimated. In contrast, PIV introduces large errors in the Z- component of the displacement field in cases where out-of-plane displacements are relatively small (less than 10% of the in-plane displacements) or negligible, i.e. when tractions are exerted purely along the X-axis. In other words, the large errors in the recovered orientation are mainly caused by the errors in the elevation angle, which in turn depends on the Z-component of the displacements. This noise in the estimated out-of-plane displacements is amplified during calculation of the traction field, affecting not only to the direction but also to the magnitude of the retrieved tractions. Note that while FFD-based estimations are not completely free of this effect, they are affected to a lesser extent, thus providing a more robust solution for the recovery of in-plane tractions.

As it was previously observed qualitatively, FFD and PIV algorithms showed similar performance for all the tested conditions when out-of-plane tractions were applied. In the case of tractions applied along the Z-axis, errors on ‘on-axis’ tractions for both methods are on average larger than those shown by FFD for tractions applied along the X-axis. In contrast, the angular errors are about 4.5° on average for both methods, implying reliable direction recovery and the introduction of small non-zero ‘off-axis’ traction components (in this case, in the X- and Y-directions; see also S7–S9 Figs).

#### Shape recovery of the stress footprint

We also characterized the shape of the stress footprint by quantifying the size of the segmented area and how much it resembles the simulated circular patch. Table 2 summarizes our results. Stress footprints recovered after FFD have an almost constant circularity value above 0.9 in a 78% of the evaluated condition, which indicates good shape recovery of the stress footprint. In contrast, PIV shows worse shape preservation with a large variability of the circularity metric, providing values above 0.9 only in 11% of the test conditions. As for the errors in the size of the segmented area, while FFD shows comparable or improved results for in-plane traction fields, both algorithms perform equally well on average for tractions applied along the Z-axis.

**Table 2:** Quantitative performance evaluation of the segmented stress footprint shape for FFD and PIV algorithms within a TFM framework.

		FFD									PIV								
		10um			6um			4um			10um			6um			4um		
		15%	10%	5%	15%	10%	5%	15%	10%	5%	15%	10%	5%	15%	10%	5%	15%	10%	5%
**Load along X-axis**	**Area Error (%)**	*19*.*07 ± 3*.*48*	*18*.*61 ± 4*.*27*	*21*.*56 ± 3*.*93*	*20*.*01 ± 4*.*4*	*17*.*47 ± 5*.*26*	*20*.*54 ± 7*.*81*	*14*.*85 ± 2*.*3*	*16*.*68 ± 4*.*1*	*11*.*25 ± 3*.*35*	*58*.*52 ± 32*.*24*	*29*.*5 ± 9*.*66*	*23*.*3 ± 6*.*75*	*32*.*91 ± 13*.*4*	*26*.*73 ± 12*.*55*	*27*.*47 ± 11*.*46*	*17*.*86 ± 5*.*67*	*19*.*82 ± 6*.*84*	*-*
	**Circularity**	*0*.*91 ± 0*.*01*	*0*.*9 ± 0*.*01*	*0*.*89 ± 0*.*02*	*0*.*93 ± 0*.*01*	*0*.*92 ± 0*.*02*	*0*.*92 ± 0*.*02*	*0*.*94 ± 0*.*02*	*0*.*93 ± 0*.*02*	*0*.*92 ± 0*.*03*	*0*.*79 ± 0*.*19*	*0*.*72 ± 0*.*08*	*0*.*61 ± 0*.*12*	*0*.*84 ± 0*.*08*	*0*.*75 ± 0*.*12*	*0*.*72 ± 0*.*13*	*0*.*84 ± 0*.*12*	*0*.*84 ± 0*.*1*	*-*
**Load along Z-axis**	**Area Error (%)**	*14*.*78 ± 2*.*73*	*18*.*58 ± 4*.*16*	*44*.*15 ± 14*.*03*	*23*.*2 ± 2*.*72*	*14*.*3 ± 7*.*53*	*49*.*55 ± 5*.*03*	*7*.*27 ± 1*.*92*	*13*.*14 ± 6*.*18*	*23*.*36 ± 14*.*03*	*20*.*08 ± 5*.*83*	*23*.*71 ± 9*.*97*	*37*.*09 ± 11*.*17*	*19*.*25 ± 4*.*83*	*20*.*49 ± 10*.*06*	*37*.*33 ± 12*.*86*	*16*.*45 ± 5*.*94*	*27*.*82 ± 8*.*19*	*34*.*57 ± 21*.*56*
	**Circularity**	*0*.*91 ± 0*.*01*	*0*.*88 ± 0*.*02*	*0*.*69 ± 0*.*12*	*0*.*94 ± 0*.*01*	*0*.*91 ± 0*.*02*	*0*.*93 ± 0*.*03*	*0*.*95 ± 0*.*02*	*0*.*94 ± 0*.*01*	*0*.*88 ± 0*.*06*	*0*.*81 ± 0*.*06*	*0*.*65 ± 0*.*17*	*0*.*6 ± 0*.*1*	*0*.*86 ± 0*.*04*	*0*.*83 ± 0*.*06*	*0*.*67 ± 0*.*17*	*0*.*94 ± 0*.*02*	*0*.*95 ± 0*.*03*	*0*.*77 ± 0*.*12*

Evaluation conditions include 20 realizations with loads with magnitudes of 15%, 10% and 5% of the substrate Young’s modulus, aligned with the X and Z Cartesian direction and distributed over a circular area of 10μm, 6μm and 4μm diameter. Errors in the recovered stress footprint area given in percentage. Circularity ranges between 0 (very spiky and/or elongated shapes) and 1 (perfect circle).

These findings agree with the qualitative assessment of the stress footprints shown in Fig 5 for every tested condition, where it can be easily observed how FFD promotes on average a better shape recovery of the stress footprint.

### FFD-Based 3D Traction Recovery of Human Umbilical Vein Endothelial Cells on a Polyacrylamide Substrate

**Fig 8:**
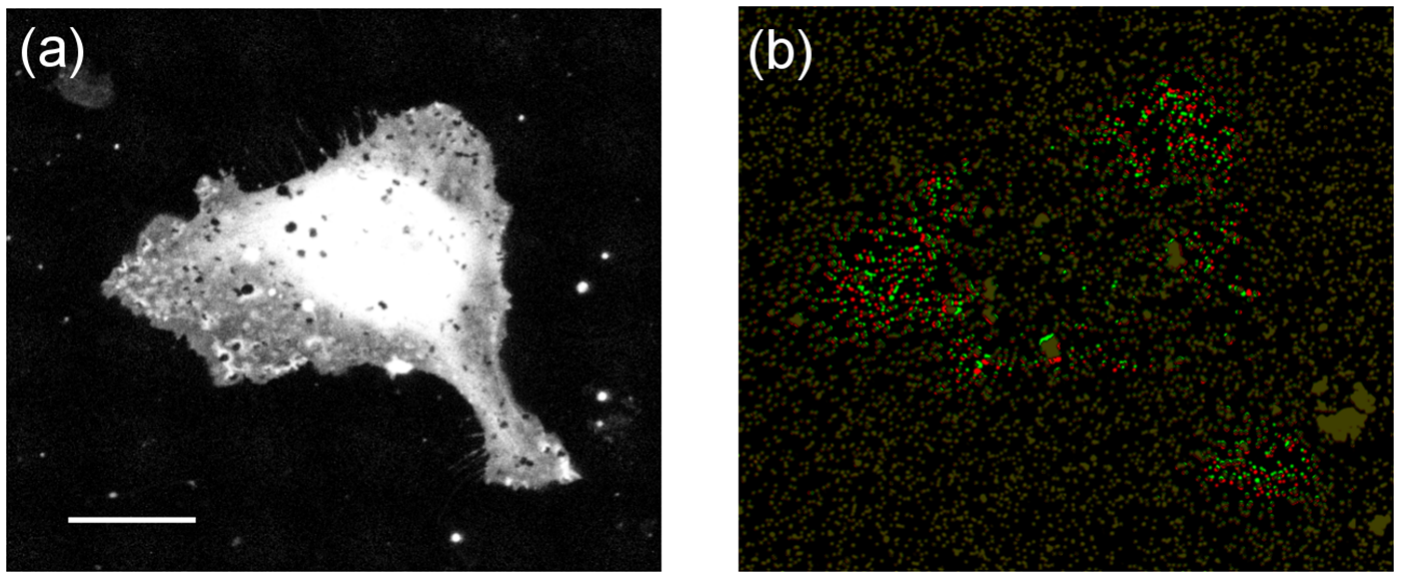
Displacements of fluorescent beads induced by a GFP-HUVEC on a 1.3 kPa PAA gel. (a) Maximum intensity projection of the fluorescent image of the HUVEC. (b) Pseudo-color image showing the fluorescent beads at the gel surface. The beads in the unstressed and stressed gel are pseudo-colored in red and green, respectively. The contrast of the pseudo-color image has been modified to highlight the areas with bead displacements. The scale bar represents 30μm.

**Fig 9:**
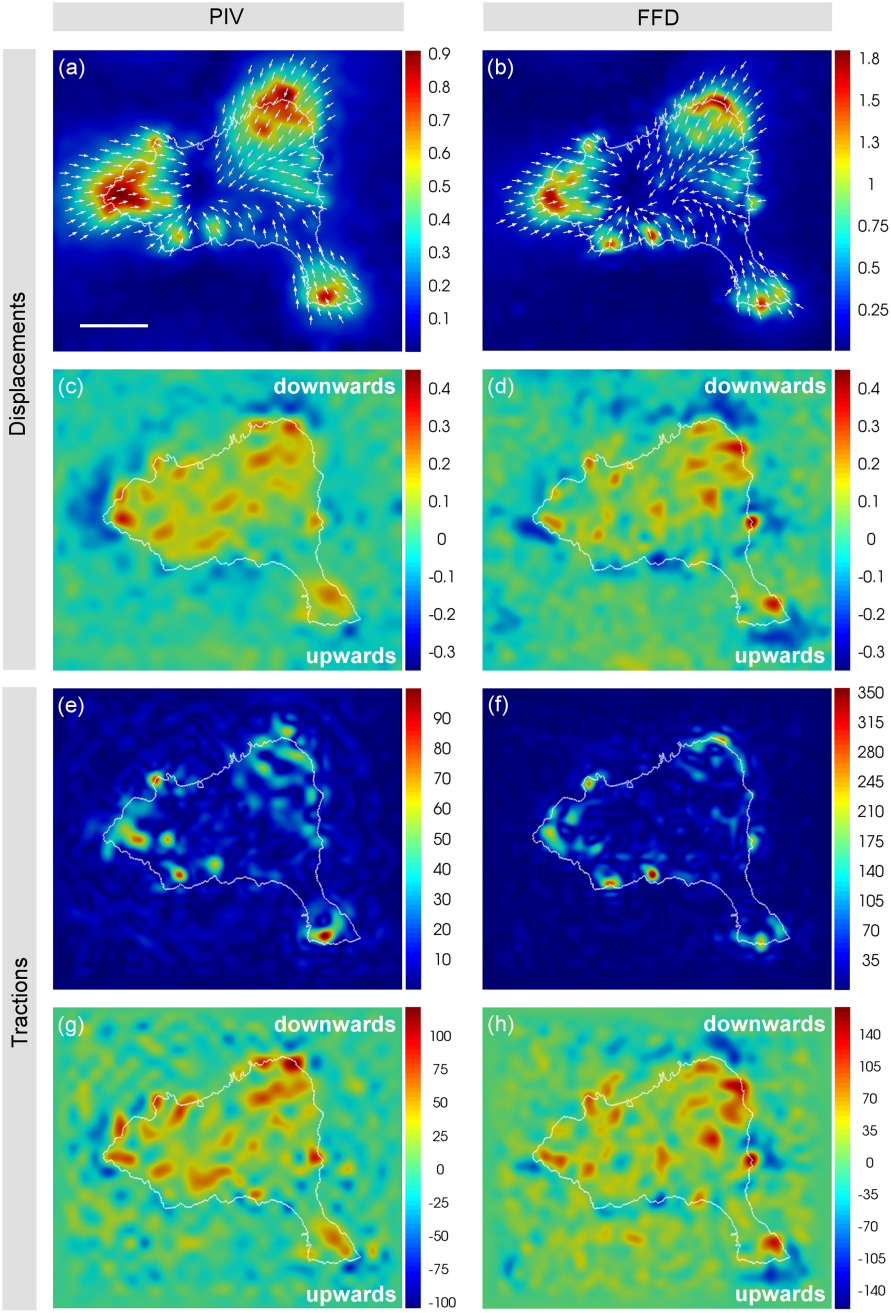
Displacement and traction fields at the gel top surface exerted by a HUVEC on a 1.3 kPa PAA gel. Magnitude (in μm) of in-plane displacements calculated by PIV (a) and FFD (b). Arrows indicate the direction of the displacements. PIV-based (c) and FFD-based (d) out-of-plane displacements (in μm). Positive and negative sign refer to downward and upward displacements respectively. Magnitude (in Pa) of in-plane (e, f) and out-of-plane (g, h) tractions obtained from PIV-based (e, g) and FFD-based (f, h) displacements. Positive and negative sign of the out-of-plane traction (g, h) indicate downward and upward traction, respectively. The cell boundary is outlined in white. The scale bar represents 30μm.

As an application, we used the presented computational methods to quantify the displacements induced by commercial human umbilical vein endothelial cells expressing Green Fluorescent Protein (GFP-HUVEC, Angio-Proteomie, Boston, MA). These cells were cultured on a polyacrylamide (PAA) hydrogel with a Young’s modulus of 1.3kPa (Fig 8a). Previously established protocols were used for the experimental setup, including the preparation of the substrates, cell culture and live cell imaging, as detailed in S1 File. Results are shown here for two different representative cells (see Figs 8 and 9 and S10 Fig).

Fig 8b shows the bead displacements induced by a HUVEC. The beads of the unstressed and stressed hydrogels have been pseudo-colored in red and green, respectively; therefore, beads are colored yellow when not displaced. Additionally, the contrast of the pseudo-color image has been modified to highlight the areas with bead displacements.

Fig 9 shows the in-plane and out-of-plane displacements and tractions at the gel top surface, based on either PIV (Fig 9a, 9c, 9e and 9g) or FFD (Fig 9b, 9d, 9f and 9h). The parameter values used to calculate the displacements and to recover the forces were the same as those used in the synthetic data, except for the grid/block size of the finest scale, which was optimized for the specific experimental settings and resulted to be 4x4x4.5μm for FFD and 5.4x5.4x6μm for PIV. The displacement field was computed from the movement of the beads in the acquired image volumes of the stressed and unstressed hydrogel. The displacement used to recover the tractions was calculated in a 4.5μm-thick gel volume below the top surface. In-plane and out-of-plane tractions were mainly localized near the cell periphery (Fig 9e, 9g, 9f and 9h). In plane tractions pointed to the cell center/nucleus, while more distal regions (with respect to cell center) with upward out-of-plane tractions were found adjacent to more proximal regions with downward out-of-plane tractions

The analysis of the calculated displacement fields reveals that PIV spreads in-plane displacements over larger areas (Fig 9a) and with magnitudes ~50% smaller than the ones provided by FFD (Fig 9b). This, in turn, negatively affects the recovered traction magnitudes (Fig 9e). Regarding the out-of-plane components, both FFD and PIV showed similar performance for displacement calculation (Fig 9c and 9d) and traction recovery (Fig 9g and 9h), being both noisier than those for the in-plane components.

## Discussion

Traction Force Microscopy (TFM) is the state-of-the-art method to calculate the tractions that cells exert on their surrounding extracellular environment. TFM is well established for cells cultured in 2D, and recent efforts have extended its use to 3D environments [29]. Independent of the selected setup, 2D or 3D, cell traction recovery is strongly affected by the errors introduced during the calculation of the substrate deformation. This often causes incorrect recovery of the traction force data leading to misinterpretations of the cell behavior. These errors are related both to the experimental set-up and the strategy used to estimate the displacement field from the movements of the fluorescent beads. For instance, low bead densities lead low spatial resolution in the displacement field estimation, causing aliasing artifacts during traction reconstruction. Consequently, moving towards reliable high resolution TFM calculation requires improving the performance of the algorithms used in the estimation of the displacement field.

In this paper, we report the use of B-spline–based FFD to calculate the local deformations of the extracellular matrix caused by cellular forces. This technique relies on a non-rigid registration of the substrate images by manipulating an underlying regular mesh. Intuitively, it can be viewed as an enhanced version of the rigid block-matching method, usually known as PIV in TFM literature. Indeed, while PIV divides the bead images in a sequence of disjoint blocks, FFD is based on a connected deformable mesh, which allows a wider range of flexible deformations. As previously mentioned, some block-matching algorithms model the displacement field within each block by high-order polynomials [16,17]. However, estimating the parameters of these models for every block is computationally expensive. In contrast, FFD has no assumptions on the underlying deformation and can be efficiently implemented. Recently, a method that relies on simultaneous iterative registration of the unstressed and stressed bead images has been applied to TFM [30]. However, this method still relies on a correlation-based block-matching algorithm and not in a fully deformable mesh as in our algorithm.

Using synthetic experiments we have compared the ability of our FFD-based algorithm to recover 3D tractions exerted by cells on the surface of a soft elastic substrate with that of the standard PIV algorithm. To that end, we have evaluated the errors of the recovered traction fields under different test conditions. In particular, we have analyzed the shape of the recovered stress footprint, along with its total force magnitude and average orientation.

Our results show that FFD outperforms PIV in a larger number of evaluated conditions. In contrast to FFD, PIV introduces large errors in recovered tractions when out-of-plane displacements are relatively small (<10%) with respect to the in-plane displacements, as in the case of pure in-plane tractions exerted on the surface of the substrate. However, these relatively large errors are only present in the Z-component of the calculated displacement field. Thus, they would not affect the displacements estimated using the PIV method within a 2D TFM experiment (i.e., estimating only X- and Y-displacements). Moreover, we found that PIV underestimates in-plane tractions, thus introducing a second source of error that propagates through traction recovery. Conversely, FFD-based traction estimations are more robust. In particular, we observe a smaller variability in the error metrics with respect to the spatial distribution of the fluorescent beads. The larger variability of the PIV-based recovered tractions is probably due to its sensitivity to the way bead images are divided into blocks, which mainly affects the out-of-plane displacements.

In contrast to scenarios in which out-of-plane displacements are small, we have observed that PIV and FFD are equally precise when out-of-plane displacements are large. We hypothesize that this is caused by the large axial elongation of the point spread function of the microscope, and especially so for those beads located close to the surface of the substrate, at distances as far as 100μm from the objective. This effect requires setting a larger mesh (block size) in FFD (PIV) algorithm along the Z-axis compared to the one used for the XY-plane. Indeed, this larger mesh/block size would behave as a low-pass filter that smoothens out the high frequency information in both FFD and PIV algorithms.

It is important to remark that the selected mesh/block size in FFD/PIV has a great impact on the overall resulting displacements. As a general rule, it should be selected for every experiment according to the bead density (provided a uniform random distribution of the beads). On one hand, large mesh/block sizes will smooth out the displacement field, resulting in an underestimation of its magnitude and a loss of the spatial resolution. On the other hand, too small mesh/block sizes will be prone to larger errors and noisier displacements. As explained previously, given that cells usually shows tractions of different magnitudes over focal adhesions of diverse sizes, we could not fit a block/mesh size independently to each condition. Instead, we empirically chose a block/mesh size suitable for most of the evaluated conditions. In particular, we found PIV to be more sensitive to the specific bead distribution in that it requires slightly larger block/mesh sizes than FFD to ensure the stability of the results. This fact implies that FFD can capture a wider range of deformations, thus better adapting to the deformations caused by the cells at multiple neighboring focal adhesions. However, to demonstrate that the described results are consistently shown when equal block/mesh sizes are used for PIV and FFD, and allow for a direct comparison of both methods in identical conditions, additional simulations were performed using a reasonable range of block/mesh sizes. The quantitative evaluation is shown in S11 and S12 Figs for in-plane and out-of-plane tractions, respectively.

Note that the simulations presented in this work are based on uniform tractions aligned with the X (in-plane) or Z (out-of-plane) Cartesian directions; therefore, they do not implicitly account for all the elevation angles observed in the traction fields exerted by cells. Nevertheless, as any traction direction results from the combination of the in-plane and out-of-plane components, the conclusions obtained from these two extreme cases (elevation angles of 0° and 90°) serve to quantitatively characterize the performance of different TFM algorithms. Moreover, they can also guide the interpretation of the results from real experiments.

As an example of application of our method, we compared the ability of both FFD and PIV to capture the displacements and tractions exerted GFP-HUVECs cultured on a polyacrylamide hydrogel. Based on the results obtained using simulated data, FFD is expected to provide results closer to reality and PIV is suspected to underestimate the magnitudes of the HUVEC-mediated displacement and traction fields, especially for the in-plane components. Indeed, this could explain the loss of weak/small traction patches in some cases, as shown in S10 Fig.

As for the traction distribution of HUVECs, three major effects can be highlighted from the results. First, HUVECs exert in-plane and out-of-plane tractions of comparable magnitude. Second, the in-plane components were located at cell periphery in agreement with previous 2D TFM experiments [31–33]. Third, out-of-plane components were mainly present close to cell periphery, showing a spatial pattern similar to the local rotational moments reported first for mouse embryo fibroblast [9] and more recently for Schwann cells [13]. Interestingly, this push-pull configuration dissents from the distribution of the out-of-plane tractions reported for other endothelial cell lines such as bovine aortic endothelial cells (BAECs) [10]. Those pull upwards at the cell periphery while pushing downwards underneath the cell centroid, suggesting nuclear compression. As proposed in [9], the origin of this discrepancy may be found in the differences between cell lines (HUVECs vs. BAECs), cell shape or the stiffness of the hydrogel where cells were cultured (~1.3kPa in this study vs. ~3.8kPa for previous report). However, although to our knowledge, this is the first study reporting the distribution of out-of-plane tractions exerted by HUVECs on soft substrates, these results were generated only as a proof of principle to test the applicability of the computational methods presented in this paper. They do not pretend to provide an in depth study of the tractions exerted by endothelial cells in general or HUVECs in particular.

Finally, the FFD method may also be suitable to accurately calculate full field displacements and quantify the tractions of cells embedded in a more physiological, fibrillar matrix such as collagen without the need to embed fluorescent beads, which could alter the matrix mechanical properties. This could be achieved by fluorescently labeling the fibrils. In addition, FFD could lead to a more efficient computation of the deformation gradient tensor in the Strain-Stress–based TFM approach [34] as the expressions for the derivatives are known in closed form due to its B-spline–based modeling of the displacements.

In summary, the recovery of cell tractions is highly affected by the method used to calculate the displacement field. The systematic and quantitative analysis presented in this work shows the limitations of the popular PIV method when applied to TFM experiments. Namely, it leads to an underestimation of in-plane traction magnitudes and to strong errors in the estimation of the Z-component of the displacement field when small out-of-plane tractions are present. The proposed FFD-based non-rigid image registration is a robust alternative that reduces these errors in the calculation of 2D/3D displacement fields within TFM experiments and provides a more accurate and robust traction recovery.

## Supporting Information

S1 Fig Example of a simulated TFM data set for a circular traction patch of 6μm diameter exerting a load of 10% of the Young modulus along the Z-axis. (a) Magnitude of the traction field, (b) magnitude of the displacements caused by the tractions in (a), and a simulated substrate volume containing 0.2μm fluorescent beads (c). Units of the tractions are given as percentage of the Young’s modulus. Units of the displacements are given in μm. The scale bars represent 5μm.
(TIFF)

S2 Fig Representative examples of the angular directions within the recovered stress footprints for loads along X-axis. Angular directions within the recovered stress footprints for tractions with magnitudes of 15%, 10% and 5% of the substrate Young’s modulus, aligned with the X Cartesian direction and distributed over a circular area of 10μm, 6μm and 4μm diameter. The colormap indicates the elevation angle (with respect to X-axis). Units of colorbars are given in degrees.
(TIFF)

S3 Fig Representative examples of the angular directions within the recovered stress footprints for loads along Z-axis. Angular directions within the recovered stress footprints for tractions with magnitudes of 15%, 10% and 5% of the substrate Young’s modulus, aligned with the Z Cartesian direction and distributed over a circular area of 10μm, 6μm and 4μm diameter. The colormap indicates the elevation angle. Units of colorbars are given in degrees (-90 corresponding to negative Z-axis).
(TIFF)

S4 Fig Representative example of displacement and traction fields for a 10μm size patch exerting loads along X-axis. X, Y, and Z-components of the surface displacement and traction fields for loads with magnitudes of 15%, 10% and 5% of the substrate Young’s modulus, aligned with the X Cartesian direction and distributed over a circular area of 10μm diameter. Axial sections of the displacements defined along the white cut-line are included. Units of colorbars are given in μm for displacements and as percentage of the Young’s modulus for tractions.
(TIFF)

S5 Fig Representative example of displacement and traction fields for a 6μm size patch exerting loads along X-axis. X, Y, and Z-components of the surface displacement and traction fields for loads with magnitudes of 15%, 10% and 5% of the substrate Young’s modulus, aligned with the X Cartesian direction and distributed over a circular area of 6μm diameter. Axial sections of the displacements defined along the white cut-line are included. Units of colorbars are given in μm for displacements and as percentage of the Young’s modulus for tractions.
(TIFF)

S6 Fig Representative example of displacement and traction fields for a 4μm size patch exerting loads along X-axis. X, Y, and Z-components of the surface displacement and traction fields for loads with magnitudes of 15%, 10% and 5% of the substrate Young’s modulus, aligned with the X Cartesian direction and distributed over a circular area of 4μm diameter. Axial sections of the displacements defined along the white cut-line are included. Units of colorbars are given in μm for displacements and as percentage of the Young’s modulus for tractions.
(TIFF)

S7 Fig Representative example of displacement and traction fields for a 10μm size patch exerting loads along Z-axis. X, Y, and Z-components of the surface displacement and traction fields for loads with magnitudes of 15%, 10% and 5% of the substrate Young’s modulus, aligned with the Z Cartesian direction and distributed over a circular area of 10μm diameter. Axial sections of the displacements defined along the white cut-line are included. Units of colorbars are given in μm for displacements and as percentage of the Young’s modulus for tractions.
(TIFF)

S8 Fig Representative example of displacement and traction fields for a 6μm size patch exerting loads along Z-axis. X, Y, and Z-components of the surface displacement and traction fields for loads with magnitudes of 15%, 10% and 5% of the substrate Young’s modulus, aligned with the Z Cartesian direction and distributed over a circular area of 6μm diameter. Axial sections of the displacements defined along the white cut-line are included. Units of colorbars are given in μm for displacements and as percentage of the Young’s modulus for tractions.
(TIFF)

S9 Fig Representative example of displacement and traction fields for a 4μm size patch exerting loads along Z-axis. X, Y, and Z-components of the surface displacement and traction fields for loads with magnitudes of 15%, 10% and 5% of the substrate Young’s modulus, aligned with the Z Cartesian direction and distributed over a circular area of 4μm diameter. Axial sections of the displacements defined along the white cut-line are included. Units of colorbars are given in μm for displacements and as percentage of the Young’s modulus for tractions.
(TIFF)

S10 Fig Displacement and traction fields at the gel top surface exerted by a HUVEC on a 1.3 kPa PAA gel. (a) Maximum intensity projection of the fluorescent image of a HUVEC. (b) Pseudo-color image showing the fluorescent beads at the gel surface. The beads in the unstressed and stressed gel are pseudo-colored in red and green, respectively. The contrast of the pseudo-color image has been modified to highlight the areas with bead displacements. Magnitude (in μm) of in-plane displacements calculated by PIV (c) and FFD (d). Arrows indicate the direction of the displacements. PIV-based (e) and FFD-based (f) out-of-plane displacements (in μm). Positive and negative sign refer to downward and upward displacements respectively. Magnitude (in Pa) of in-plane (g, h) and out-of-plane (i, j) tractions obtained from PIV-based (g, i) and FFD-based (h, j) displacements. Positive and negative sign of the out-of-plane traction (i, j) indicate downward and upward traction respectively. The cell boundary is outlined in white. The scale bar represents 30μm.
(TIFF)

S11 Fig Effect of the selected mesh/block size on the performance of FFD and PIV algorithms for a circular traction patch of 6μm diameter exerting a load of 10% of the Young’s modulus along the X-axis. Evaluation of the recovered total force magnitude and average orientation within the segmented stress footprint and its shape (area and circularity). Blue and yellow bars represent the average metric values (from 20 different realizations per condition) for FFD and PIV, respectively. Green markers tag the results corresponding to the mesh/block size used for the simulations described in the main text. Error metrics cannot be determined (depicted in magenta) in those cases where the algorithm fails to recover single stress footprints. The mesh/blocks sizes are given in pixels and can be converted to physical units by scaling them with the used voxel size (0.15μm in the XY plane and 0.3μm along the Z-axis).
(TIFF)

S12 Fig Effect of the selected mesh/block size on the performance of FFD and PIV algorithms for a circular traction patch of 6μm diameter exerting a load of 10% of the Young’s modulus along the Z-axis. Evaluation of the recovered total force magnitude and average orientation within the segmented stress footprint and its shape (area and circularity). Blue and yellow bars represent the average metric values (from 20 different realizations per condition) for FFD and PIV, respectively. Green markers tag the results corresponding to the mesh/block size used for the simulations described in the main text. The mesh/blocks sizes are given in pixels and can be converted to physical units by scaling them with the used voxel size (0.15μm in the XY plane and 0.3μm along the Z-axis).
(TIFF)

S1 File Experimental Methods. (DOCX)

S2 File FFD-based TFM software for 2D and 2.5D TFM. (ZIP)

S1 Table Percentage of realizations providing a single stress footprint. Evaluation conditions include 20 realizations with loads with magnitudes of 15%, 10% and 5% of the substrate Young’s modulus, aligned with the X and Z Cartesian direction and distributed over a circular area of 10μm, 6μm and 4μm diameter.
(DOCX)
