# Supplementary figures and images for: Free Form Deformation–Based Image Registration Improves Accuracy of Traction Force Microscopy
Source: PLoS One. 2015 Dec 7;10(12):e0144184. doi: 10.1371/journal.pone.0144184 (PMC4671587; doi:10.1371/journal.pone.0144184)

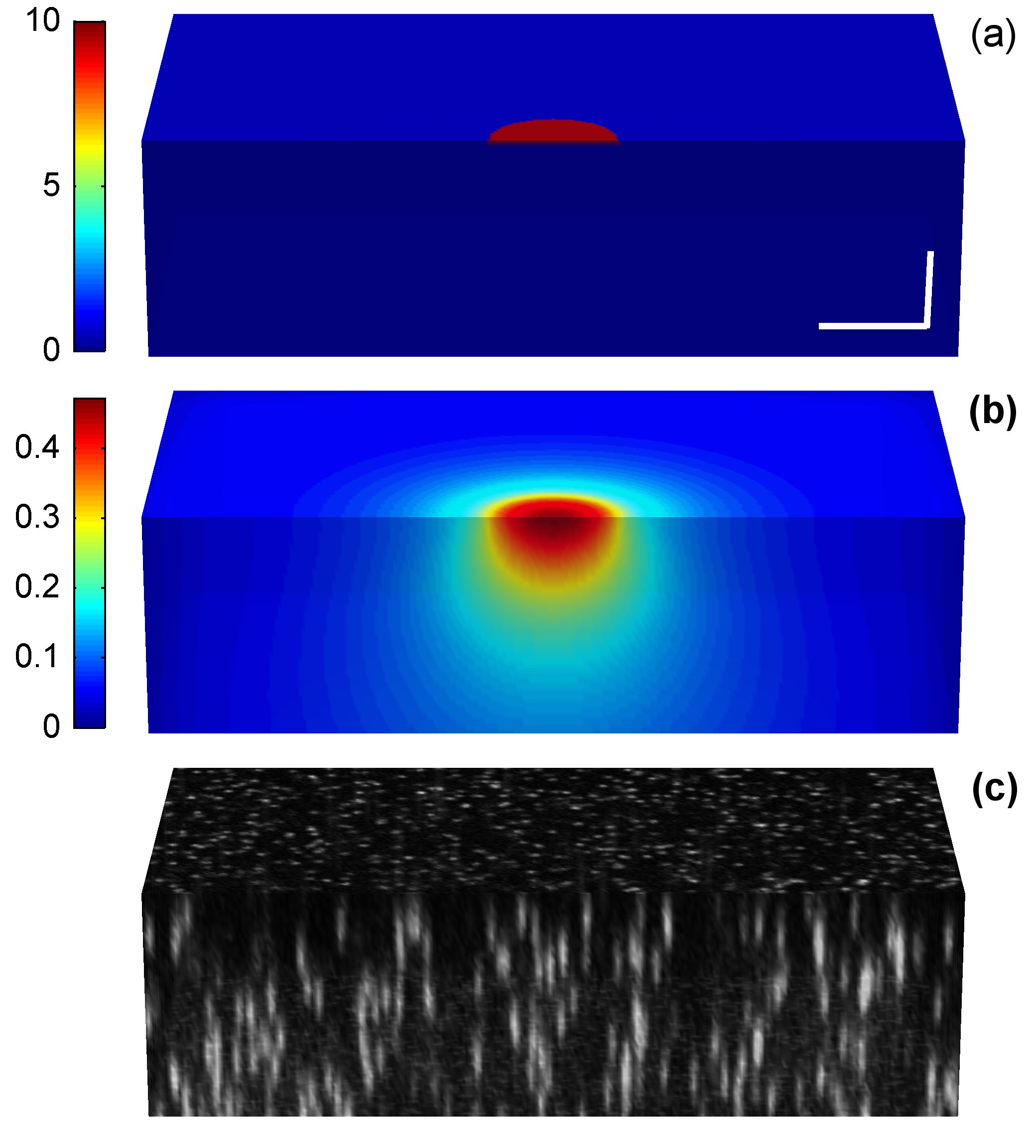

Supplement: S1 Fig — (a) Magnitude of the traction field, (b) magnitude of the displacements caused by the tractions in (a), and a simulated substrate volume containing 0.2μm fluorescent beads (c). Units of the tractions are given as percentage of the Young’s modulus. Units of the displacements are given in μm. The scale bars represent 5μm. (TIFF) [file pone.0144184.s001.tiff]

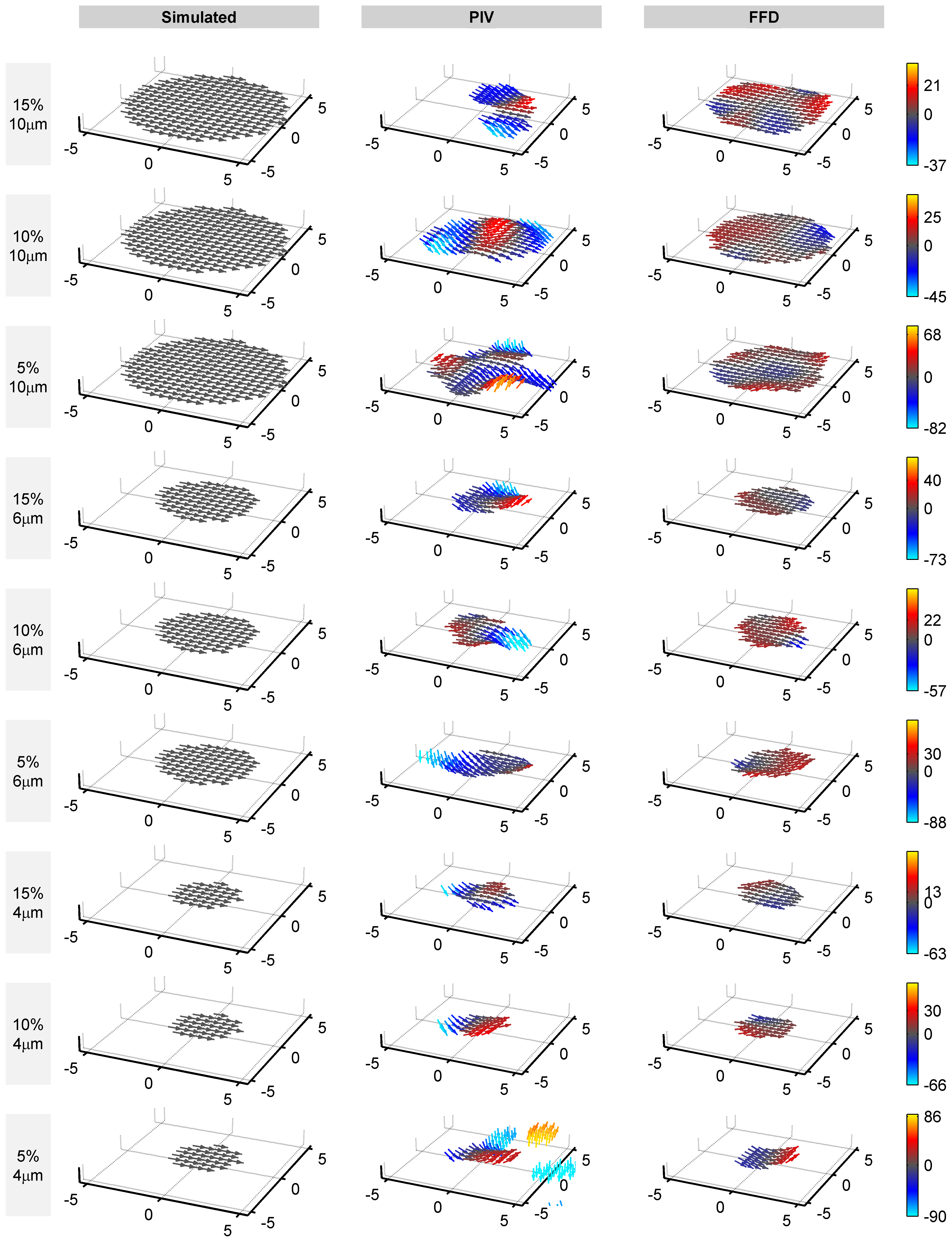

Supplement: S2 Fig — Angular directions within the recovered stress footprints for tractions with magnitudes of 15%, 10% and 5% of the substrate Young’s modulus, aligned with the X Cartesian direction and distributed over a circular area of 10μm, 6μm and 4μm diameter. The colormap indicates the elevation angle (with respect to X-axis). Units of colorbars are given in degrees. (TIFF) [file pone.0144184.s002.tiff]

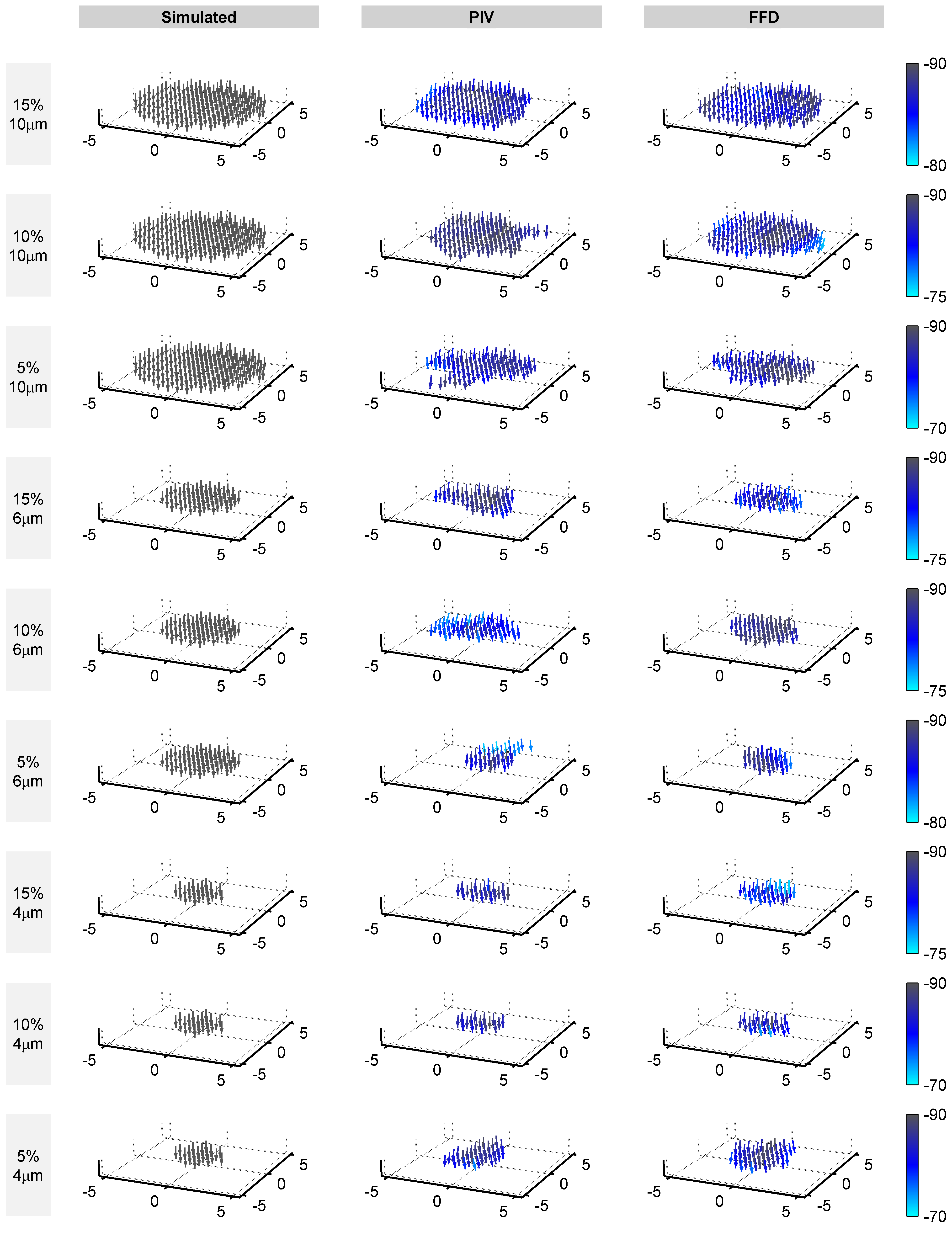

Supplement: S3 Fig — Angular directions within the recovered stress footprints for tractions with magnitudes of 15%, 10% and 5% of the substrate Young’s modulus, aligned with the Z Cartesian direction and distributed over a circular area of 10μm, 6μm and 4μm diameter. The colormap indicates the elevation angle. Units of colorbars are given in degrees (-90 corresponding to negative Z-axis). (TIFF) [file pone.0144184.s003.tiff]

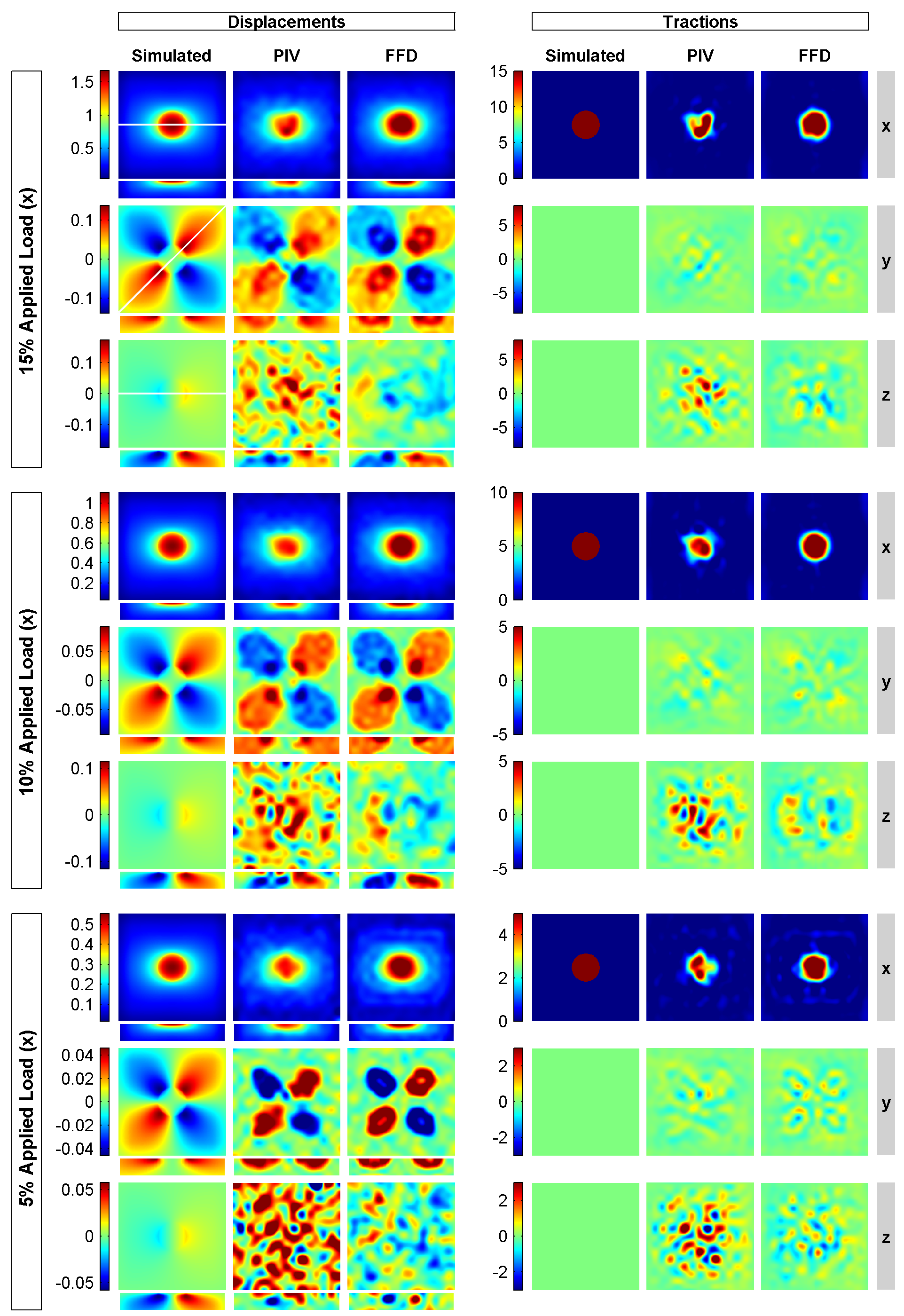

Supplement: S4 Fig — X, Y, and Z-components of the surface displacement and traction fields for loads with magnitudes of 15%, 10% and 5% of the substrate Young’s modulus, aligned with the X Cartesian direction and distributed over a circular area of 10μm diameter. Axial sections of the displacements defined along the white cut-line are included. Units of colorbars are given in μm for displacements and as percentage of the Young’s modulus for tractions. (TIFF) [file pone.0144184.s004.tiff]

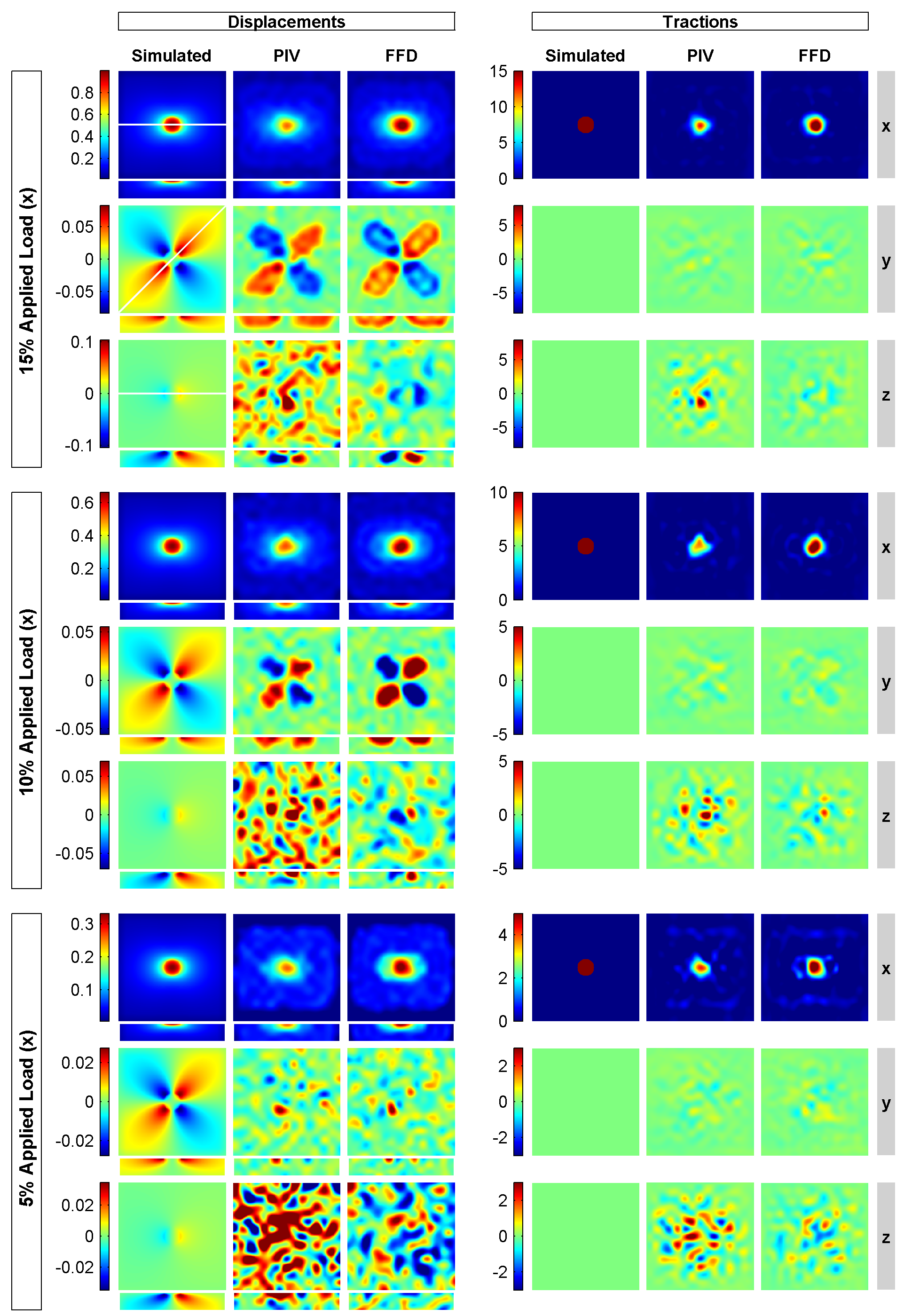

Supplement: S5 Fig — X, Y, and Z-components of the surface displacement and traction fields for loads with magnitudes of 15%, 10% and 5% of the substrate Young’s modulus, aligned with the X Cartesian direction and distributed over a circular area of 6μm diameter. Axial sections of the displacements defined along the white cut-line are included. Units of colorbars are given in μm for displacements and as percentage of the Young’s modulus for tractions. (TIFF) [file pone.0144184.s005.tiff]

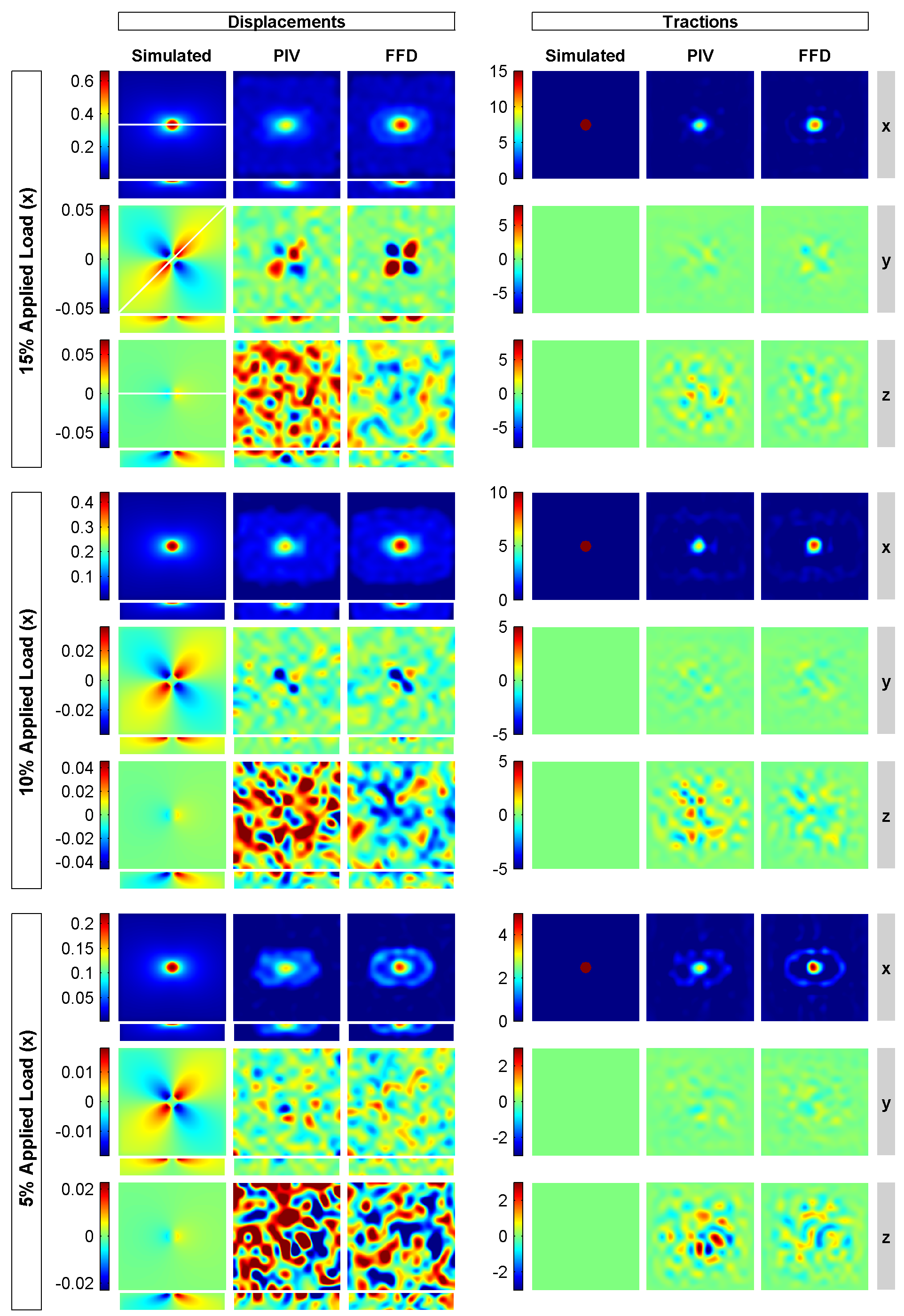

Supplement: S6 Fig — X, Y, and Z-components of the surface displacement and traction fields for loads with magnitudes of 15%, 10% and 5% of the substrate Young’s modulus, aligned with the X Cartesian direction and distributed over a circular area of 4μm diameter. Axial sections of the displacements defined along the white cut-line are included. Units of colorbars are given in μm for displacements and as percentage of the Young’s modulus for tractions. (TIFF) [file pone.0144184.s006.tiff]

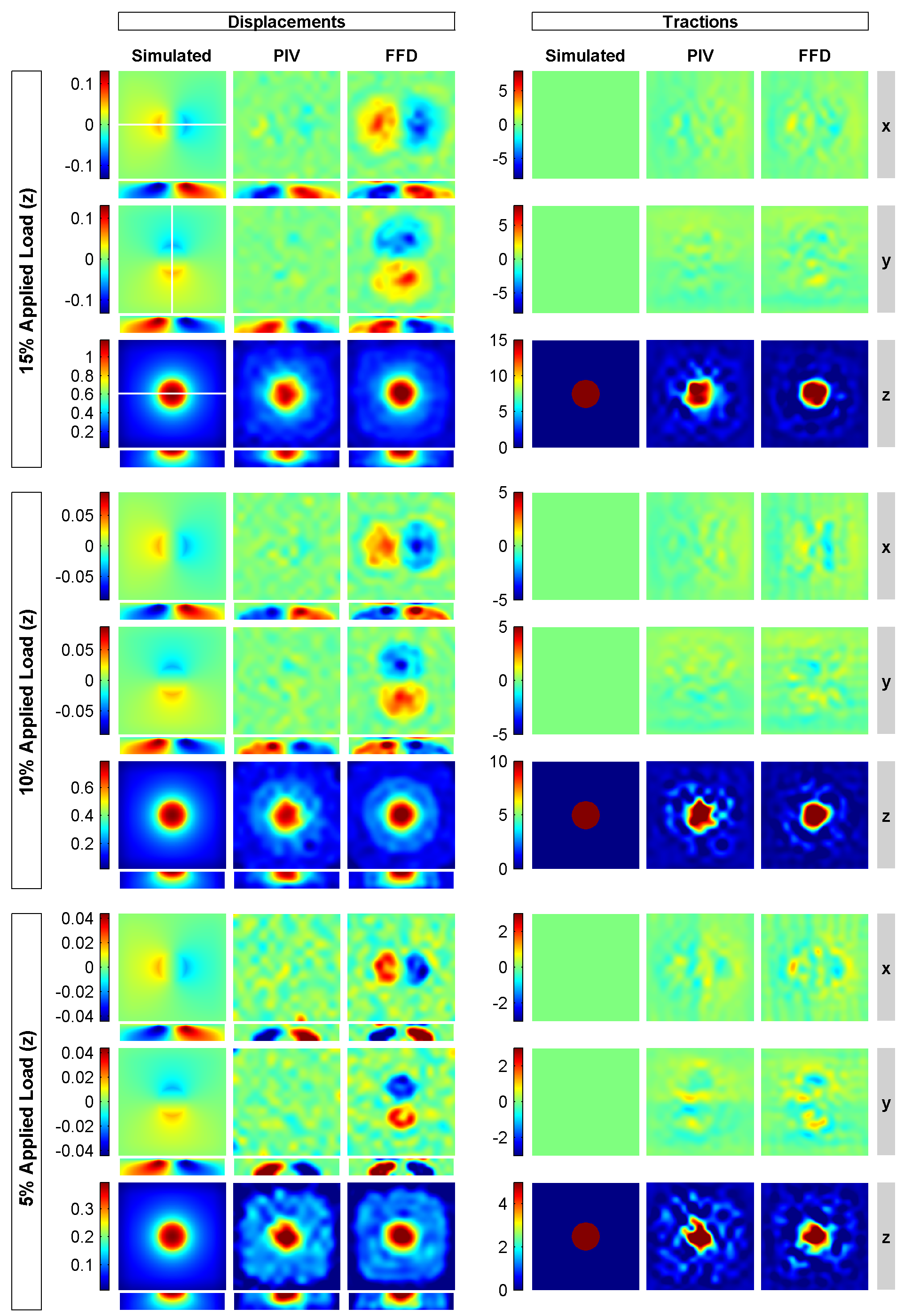

Supplement: S7 Fig — X, Y, and Z-components of the surface displacement and traction fields for loads with magnitudes of 15%, 10% and 5% of the substrate Young’s modulus, aligned with the Z Cartesian direction and distributed over a circular area of 10μm diameter. Axial sections of the displacements defined along the white cut-line are included. Units of colorbars are given in μm for displacements and as percentage of the Young’s modulus for tractions. (TIFF) [file pone.0144184.s007.tiff]

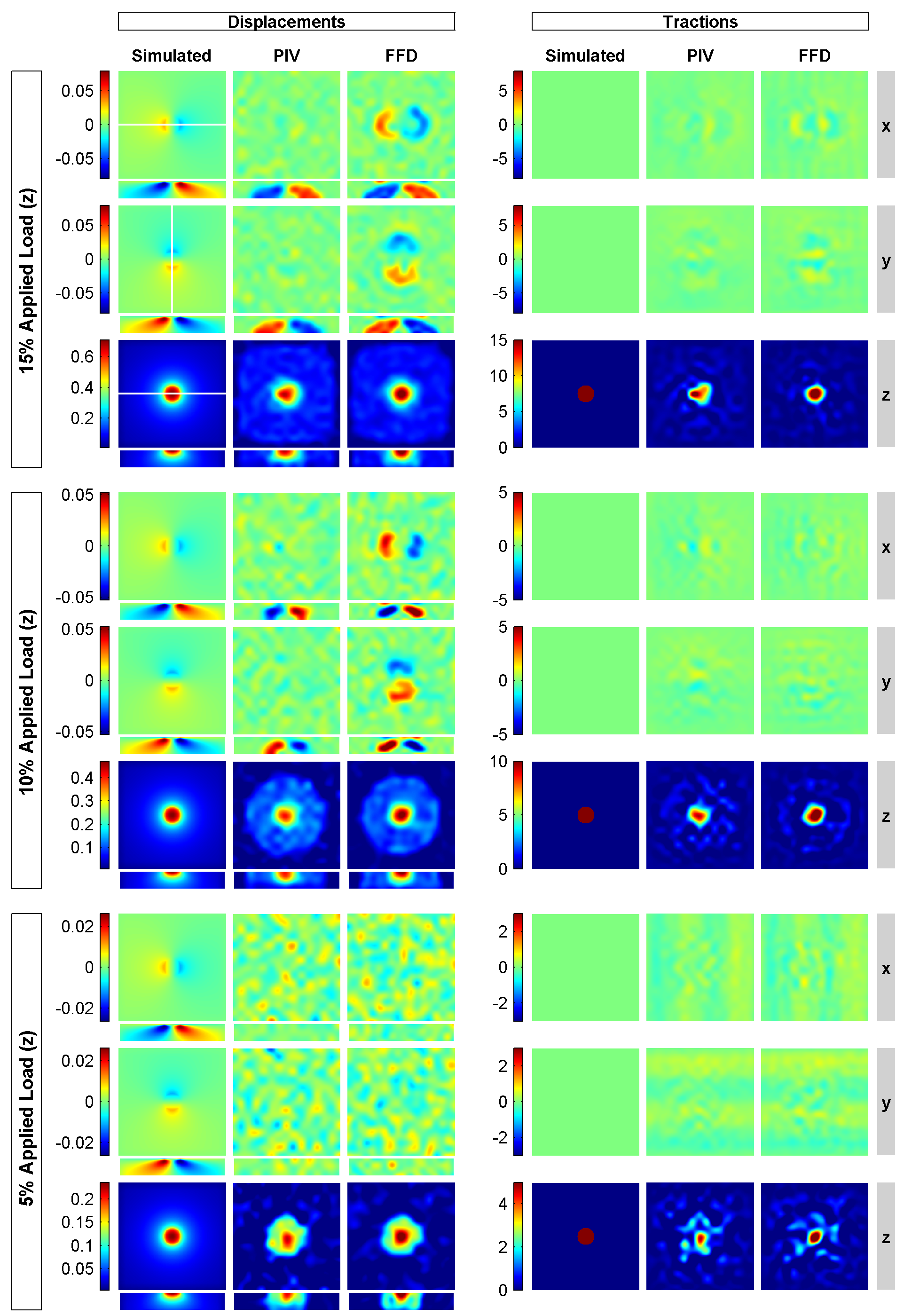

Supplement: S8 Fig — X, Y, and Z-components of the surface displacement and traction fields for loads with magnitudes of 15%, 10% and 5% of the substrate Young’s modulus, aligned with the Z Cartesian direction and distributed over a circular area of 6μm diameter. Axial sections of the displacements defined along the white cut-line are included. Units of colorbars are given in μm for displacements and as percentage of the Young’s modulus for tractions. (TIFF) [file pone.0144184.s008.tiff]

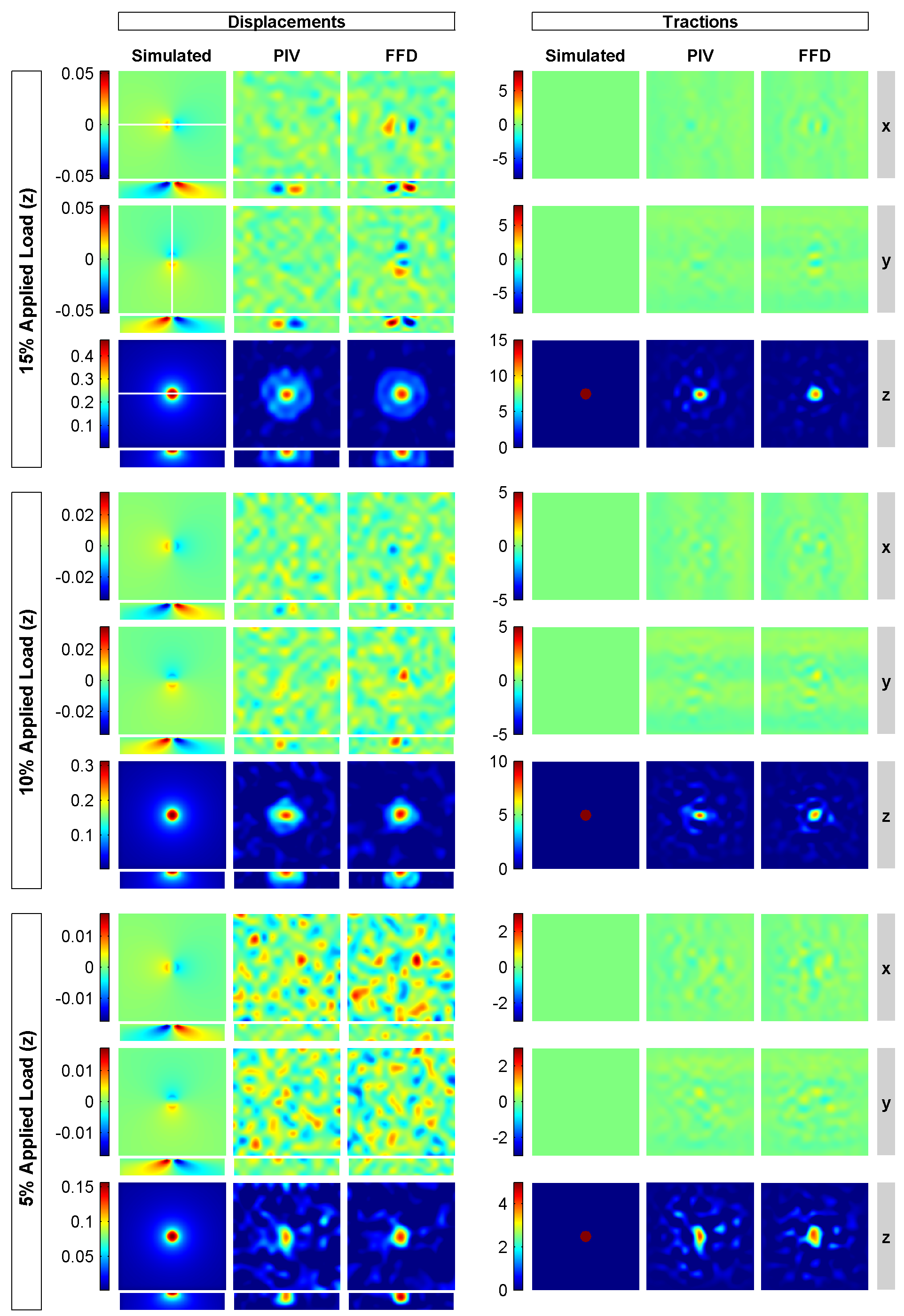

Supplement: S9 Fig — X, Y, and Z-components of the surface displacement and traction fields for loads with magnitudes of 15%, 10% and 5% of the substrate Young’s modulus, aligned with the Z Cartesian direction and distributed over a circular area of 4μm diameter. Axial sections of the displacements defined along the white cut-line are included. Units of colorbars are given in μm for displacements and as percentage of the Young’s modulus for tractions. (TIFF) [file pone.0144184.s009.tiff]

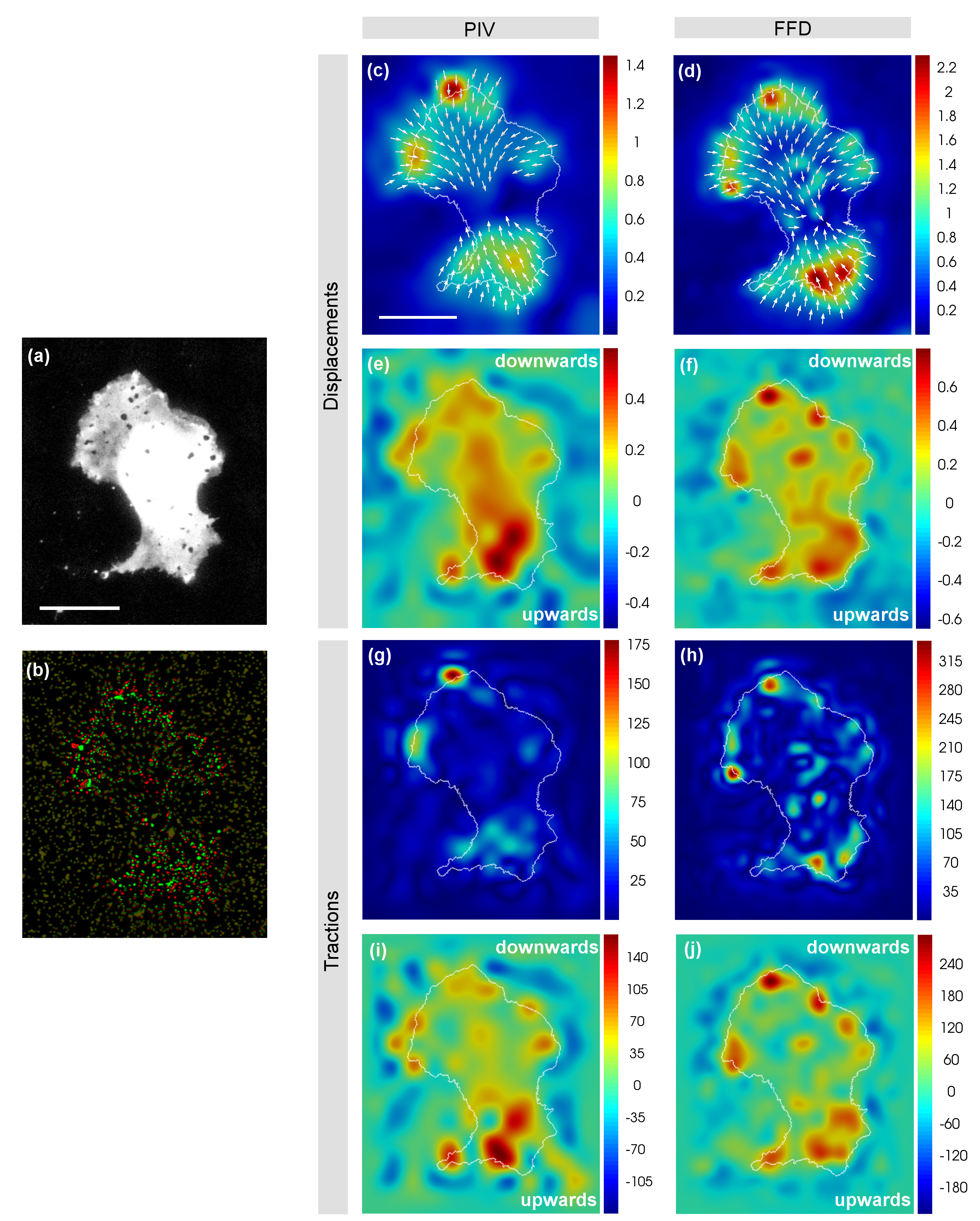

Supplement: S10 Fig — (a) Maximum intensity projection of the fluorescent image of a HUVEC. (b) Pseudo-color image showing the fluorescent beads at the gel surface. The beads in the unstressed and stressed gel are pseudo-colored in red and green, respectively. The contrast of the pseudo-color image has been modified to highlight the areas with bead displacements. Magnitude (in μm) of in-plane displacements calculated by PIV (c) and FFD (d). Arrows indicate the direction of the displacements. PIV-based (e) and FFD-based (f) out-of-plane displacements (in μm). Positive and negative sign refer to downward and upward displacements respectively. Magnitude (in Pa) of in-plane (g, h) and out-of-plane (i, j) tractions obtained from PIV-based (g, i) and FFD-based (h, j) displacements. Positive and negative sign of the out-of-plane traction (i, j) indicate downward and upward traction respectively. The cell boundary is outlined in white. The scale bar represents 30μm. (TIFF) [file pone.0144184.s010.tiff]

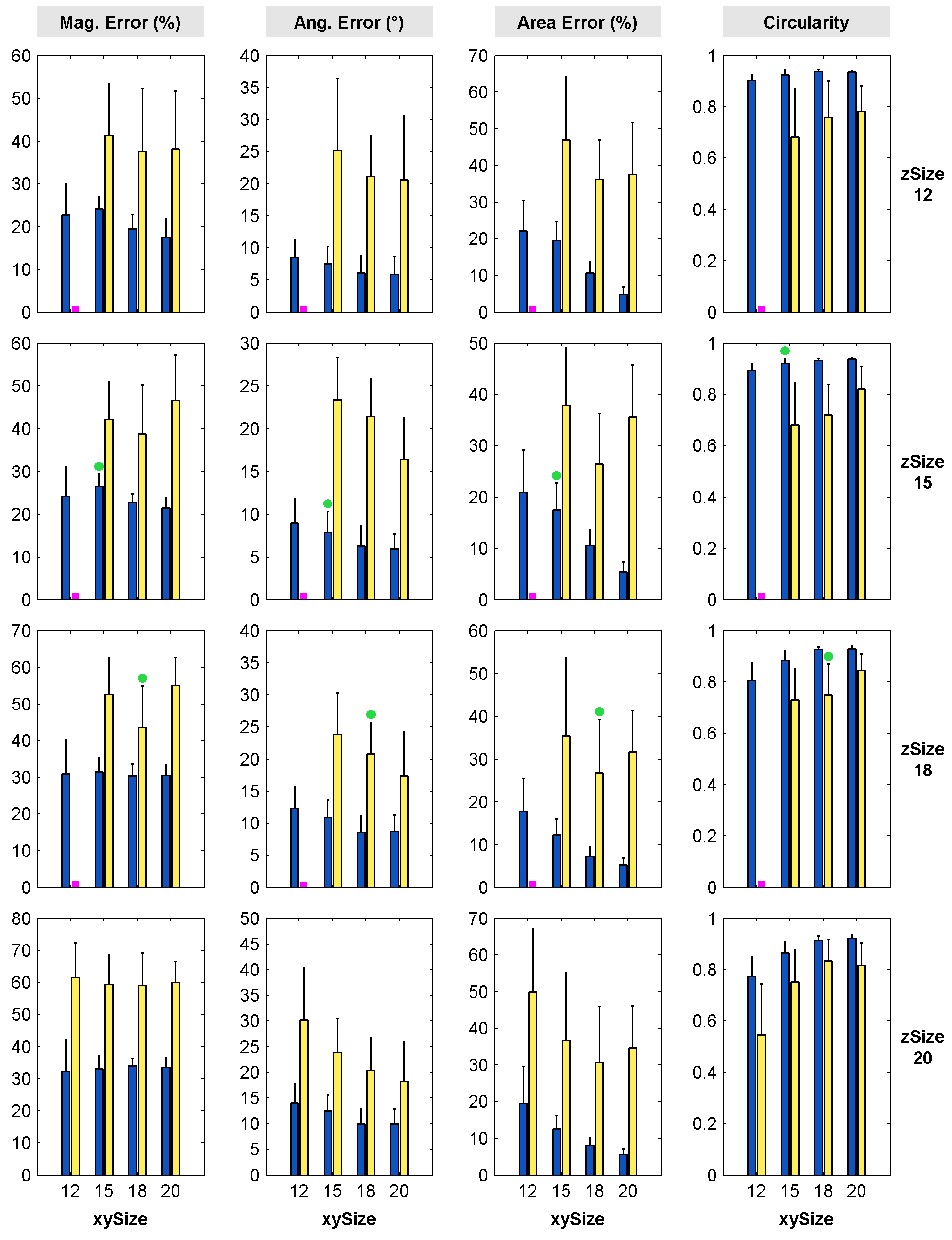

Supplement: S11 Fig — Evaluation of the recovered total force magnitude and average orientation within the segmented stress footprint and its shape (area and circularity). Blue and yellow bars represent the average metric values (from 20 different realizations per condition) for FFD and PIV, respectively. Green markers tag the results corresponding to the mesh/block size used for the simulations described in the main text. Error metrics cannot be determined (depicted in magenta) in those cases where the algorithm fails to recover single stress footprints. The mesh/blocks sizes are given in pixels and can be converted to physical units by scaling them with the used voxel size (0.15μm in the XY plane and 0.3μm along the Z-axis). (TIFF) [file pone.0144184.s011.tiff]

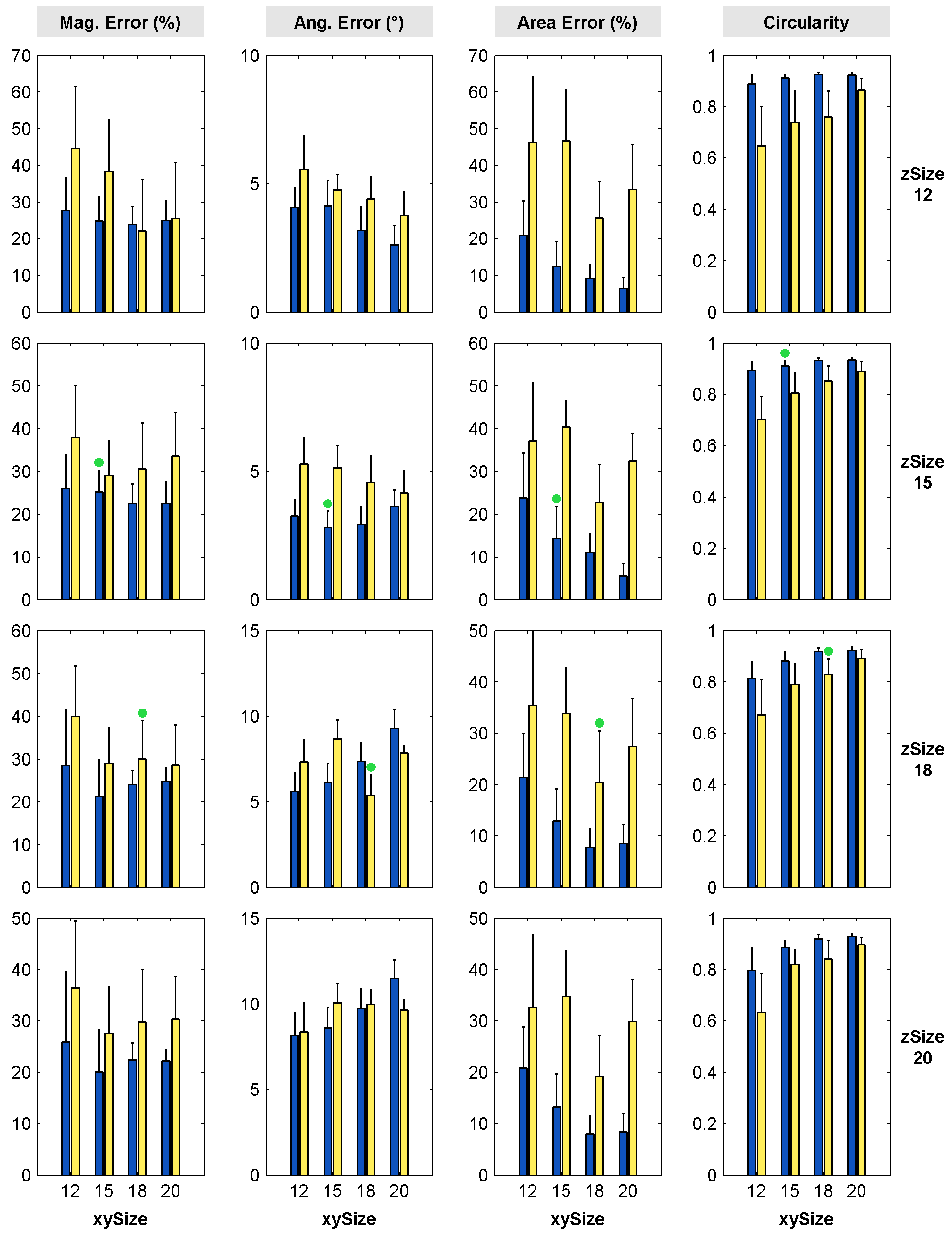

Supplement: S12 Fig — Evaluation of the recovered total force magnitude and average orientation within the segmented stress footprint and its shape (area and circularity). Blue and yellow bars represent the average metric values (from 20 different realizations per condition) for FFD and PIV, respectively. Green markers tag the results corresponding to the mesh/block size used for the simulations described in the main text. The mesh/blocks sizes are given in pixels and can be converted to physical units by scaling them with the used voxel size (0.15μm in the XY plane and 0.3μm along the Z-axis). (TIFF) [file pone.0144184.s012.tiff]
