# Supplementary material for: Free Form Deformation–Based Image Registration Improves Accuracy of Traction Force Microscopy
Source: PLoS One. 2015 Dec 7;10(12):e0144184. doi: 10.1371/journal.pone.0144184 (PMC4671587; doi:10.1371/journal.pone.0144184)
Supplement: S1 File — (DOCX) [file pone.0144184.s013.docx]

**Supplementary Methods**

**Fabrication of elastic substrates**

*Activation of glass bottom-petri dishes*

Polyacrylamide (PAA) hydrogels of controlled mechanical properties were fabricated on the surface of functionalized glass bottom-petri dishes (35mm dish with 14mm glass window; MatTek Corp.), using a slightly modified existing protocol [1]. Briefly, the glass bottom of the plates were covered with 200μl of 3-aminopropyltrimethoxy-silane to increase their roughness. After 5 minutes, the bottom glasses were extensively rinsed with distilled water and air-dried. Then, 200μl of 1% glutaraldehyde was applied onto their surface for 30 min to provide a sticky surface, which facilitates the immobilization of the PAA substrates. The bottom glasses were washed and soaked again in distilled water and air-dried.

*Manufacturing of polyacrylamide films*

To produce PAA hydrogels, a solution of 40% acrylamide (Bio-Rad) and 2% bis-acrylamide (Bio-Rad) was combined with ultra pure water. Depending on the acrylamide-bis ratio, substrates with elastic moduli between ~0.1kPa and ~100kPa can be obtained. Here, a dilution with 5% of acrylamide and 0.1% of bis was used, which produced gels with a Young’s modulus of 1.3kPa. Next, ammonium persulfate (Bio-Rad) and TEMED (Bio-Rad) were added to the mixture to initiate polymerization. Immediately afterwards, fluorescent polystyrene microbeads (0.2μm in diameter, λ_em_=605nm, carboxylate-modified, Molecular Probes) in a suspension (2% solids) were mixed exhaustively with the polyacrylamide solution in a volume ratio of 1:60. Droplets of 18μl of the final solution were pipetted onto the center of the activated glass bottom-petri dishes and were carefully covered by Sigmacote (Sigma) treated circular coverslips (12mm diameter) to flatten the PAA hydrogels and make their thickness even. The petri dishes were turned upside down during the polymerization step to allow the fluorescent microbeads move towards the gel’s surface by gravity. Following polymerization, the coverslips were detached from the hydrogel surface using a pair of tweezers and the PAA substrates were washed. Finally, they were immersed into 2ml of cell culture medium to achieve the same optical properties as in a real TFM experiment.

**PAA Gel functionalization**

PAA gel functionalization was done with human plasma fibronectin (Merck Millipore) as described in [1]. Briefly, PAA gels were covered with 200μl solution of 0.5mg/ml Sulfo-SANPAH solution with 0.5% DMSO in 50mM HEPES. Hydrogels were functionalized (binding-competent) by irradiation at 10cm with a UV lamp (365nm), twice for 20 min. Sulpho-SANPAH-treated PAA gels were then incubated overnight with a PBS solution containing 10μg/ml human fibronectin to enable integrin-dependent cell adhesion.

**Cell culture**

Commercial Green Fluorescent Protein expressing human umbilical vein endothelial cells (GFP-HUVEC, Angio-Proteomie, Boston, MA) were cultured at 37° in fully supplemented Endothelial Cell Growth Medium (EGM2 BulletKit™, Lonza) in an atmosphere of 5% CO2. About 6000 cells (passages 4-6) per gel were plated on fibronectin-coated PAA gels and allowed to adhere and spread at 37° overnight before recording.

**Live cell imaging**

Three-dimensional image stacks of the PAA gels with GFP-HUVEC cells were acquired using a 40x 0.95 numerical aperture (NA) air objective mounted in an Olympus FV1000-IX81 Laser Scanning Confocal microscope equipped with a motorized, programmable stage; and an incubator chamber to maintain a controlled environment.

Z-stacks of cells and fluorescent beads were acquired at multiple locations of the stressed hydrogel with 0.265μm pixel size in the XY plane and 0.3μm along the Z axis. The location of each imaged cell was recorded. For each imaged cell, image volumes of 212μm x 212μm x 20μm containing fluorescent beads were acquired.

After imaging, several drops of 5% sodium dodecyl sulphate (SDS) solution were added to the culture medium to lyse and release the cells from the PAA substrate. Once the cells were completely removed and the gel had returned to the unstrained state, new Z-stacks of the fluorescent beads were taken at the previously recorded locations.

**References**

1. Tse JR, Engler AJ. Preparation of hydrogel substrates with tunable mechanical properties. Curr Protoc Cell Biol. 2010;Chapter 10: Unit 10.16. doi:10.1002/0471143030.cb1016s47
