# Supplementary material for: Free Form Deformation–Based Image Registration Improves Accuracy of Traction Force Microscopy
Source: PLoS One. 2015 Dec 7;10(12):e0144184. doi: 10.1371/journal.pone.0144184 (PMC4671587; doi:10.1371/journal.pone.0144184)
Supplement: S2 File — (ZIP) [file pone.0144184.s014.zip › S2_File/readmeFirst.pdf]

TFMcalc

General Description

This software package estimates the 2D/3D tractions exerted by cells laying on a biomimetic substrate. This is done by measuring the displacement of fluorescent beads embedded in the substrate, which serve as tracking points of the deformation caused by the tractions.

The TFMcalc software allows the user to select a Free Form Deformation (FFD) –based image registration method to calculate the 2D/3D displacement field. This method is capable of modeling a wide range of local deformations.

Once the displacements are computed, the 2D/3D cell tractions are recovered by Tikhonov regularized inversion of the elasticity problem in the Fourier domain. Specifically, an integral Boussinesq analytical equation is used as Green function. For more details on the integral Boussinesq analytical equation, see:

- Jorge-Peñas A, Muñoz-Barrutia A, de-Juan-Pardo EM, Ortiz-de-Solorzano C. Validation tool for traction force microscopy. Comput Methods Biomech Biomed Engin. 2014; 37–41. doi:10.1080/10255842.2014.903934

- Huang J, Peng X, Qin L, Zhu T, Xiong C, Zhang Y, et al. Determination of cellular tractions on elastic substrate based on an integral Boussinesq solution. J Biomech Eng. 2009;131: 061009. doi:10.1115/1.311876

Assumptions

The software requires the bead images of the stressed substrate (when the cell is adhered and exerting tractions) and the relaxed substrate (after removing the cell) as inputs. The TFMcalc package assumes that these images have been already corrected for the small translational shifts/errors caused during the repositioning of the microscope's stage. Additionally, for 2.5D TFM experiments (i.e. estimation of 3D tractions from 2D cells), it is assumed that the first XY-plane of the acquired Z-stack (bead image volumes) shows the beads located at the surface of the substrate.

Required Additional Software

- Matlab, including the Image Processing and Signal Processing toolboxes
- dipLib / dipImage Library for Matlab: Freely available at http://www.diplib.org/main
- elastix software: Freely available at http://elastix.isi.uu.nl/index.php

Limitations

FFD –based displacement field calculation is performed by Elastix (see required additional software). Elastix is a command-line software and thus, some scripts are required to run it from Matlab. For a Windows machine it will require a .bat script, while a .sh script will be used in Mac. The current version of the TFMcalc software has been only tested on a Windows machine and thus, just the required .bat files are included in the package. To run the TFMcalc software on a Mac, the user would have to generate a .sh file equivalent to the .bat script provided.

TFMcalc installation and running

- Copy the folder TFMcalc to the desired directory.
- To launch the software, run the TFMcalc_launch.m file. This file could be stored in any directory that could be different from the one where the TFMcalc folder is located.
- The TFMcalc_launch.m file asks the user for all the parameters needed and run the general TFM workflow. See below for more details.
- It is assumed that the required additional software has been already installed.
- TFMcalc requires some external Matlab functions. These functions have been included in the package with their corresponding license files. Additionally, they can be found at:
  - inpaint_nans.m: http://www.mathworks.com/matlabcentral/fileexchange/4551-inpaint-nans
  - inpaint_nans3.m: http://www.mathworks.com/matlabcentral/fileexchange/21214-inpainting-nan-elements-in-3-d
  - nrrdread.m: http://www.mathworks.com/matlabcentral/fileexchange/34653-nrrd-format-file-reader
  - window2.m: http://www.mathworks.com/matlabcentral/fileexchange/43827-two-dimensional-window-generator--2d-window-
  - normscorrn.m: http://vision.ucsd.edu/~pdollar/toolbox/doc/

User parameters: TFMcalc_launch.m

Parameters related to the installation and working directory:

- **pathParam.installationPath**

  The full path of the directory where the TFMcalc folder has been stored.
  For example, if the directory 'myDir' containing the TFMcalc folder is located in 'C:\' (in Windows OS), then pathParam.installationPath ='C:\myDir\TFMcalc'

- **pathParam.readPath.stressed**

  The full path of the directory where the bead image of the stressed gel (when the cell is adhered and exerting tractions) is stored. For example, if the directory 'myInputImages' containing the bead image is located in 'C:\' (in Windows OS), then pathParam.readPath.stressed='C:\myInputImages'

- **pathParam.readFile.stressed**

  The name and extension of the bead image of the stressed gel (when the cell is adhered and exerting tractions).Only .tiff and .tif formats are supported.

- **pathParam.readPath.relaxed**

  The full path of the directory where the bead image of the relaxed gel (after removing the cell) is stored. For example, if the directory 'myInputImages' containing the bead image is located in 'C:\' (in Windows OS), then pathParam.readPath.stressed='C:\myInputImages'

- **pathParam.readFile.relaxed**

  The name and extension of the bead image of the stressed gel (after removing the cell).Only .tiff and .tif formats are supported.

- **pathParam.savePath**

  The full path of the directory where the results will be stored.
  For example, if we want to save the results in the directory 'myOutputDir' located in 'C:\' (in Windows OS), then pathParam.installationPath ='C:\myOutputDir'.

- **pathParam.saveFileName**

  The name (without extension) of the .mat file that will contain all the results generated by the TFMcalc software.

General Parameters:

- **gParam.dim**

  Set this parameter to 2 for 2D TFM experiments, where only the tangential (in-plane) tractions are computed. Set this parameter to 2.5 for 2.5D TFM experiments, where both the tangential (in-plane) and normal (out-of-plane) tractions exerted by a cell on a linear elastic substrate are computed.

- **gParam.xyResolution**

  In-plane pixel/voxel size of the bead images. Units in microns.

- **gParam.zResolution**

  Out-of-plane voxel size of the bead images. Units in microns. This parameter is only needed if analyzing a 2.5D TFM experiment.

Parameters related to the Displacement Field Calculation:

- **dispParam.dispMethod**

  Method that will be used to calculate the displacement field from the bead images. Currently available options: FFD and block-matching –based PIV.

Parameters for FFD-based Displacements:

- **dispParam.ffd.meshSize.xy**

  The FFD algorithm will be run within a 3-level multiscale scheme. This parameter specifies the lateral (in-plane) size of the FFD mesh for each scale. The size of the mesh has to be provided in pixels. The scales are sorted as follows: dispParam.ffd.meshSize.xy=[coarsestScaleSize, finerScaleSize, finestScaleSize].

- **dispParam.ffd.meshSize.z**

  The FFD algorithm will be run within a 3-level multiscale scheme. This parameter specifies the normal (out-of-plane) size of the FFD mesh for each scale. The size of the mesh has to be provided in pixels. This parameter is only needed if analyzing a 2.5D TFM experiment. The scales are sorted as follows: dispParam.ffd.meshSize.z=[coarsestScaleSize, finerScaleSize, finestScaleSize].

- **dispParam.ffd.optimMethod**

  Optimization method to be used during the image registration of bead images. Currently, two possibilities are provided: quasi-Newton Limited-memory Broyden-Fletcher-Goldfarb-Shanno (quasi-Newton LBFGS) and Adaptive Stochastic Gradient Descendent (ASGD). For more information on these methods, see the documentation of Elastix.

- **dispParam.ffd.iterNum**

  Maximum number of iterations for each scale during the optimization method. The scales are sorted as follows: dispParam.ffd.interNum=[coarsestScaleMaxIter, finerScaleMaxIter, finestScaleMaxIter]. The quasi-Newton LBFGS method is able to stop before reaching the maximum number of iterations. However, the ASGD method always run until the maximum number of iterations is reached. As rule of thumb, the larger number of iterations, the more accurate results can be obtained (but it requires a longer execution time). For more information on the optimization methods, see the documentation provided with Elastix.

- **dispParam.ffd.evalSampleNum**

  Number of pixels/voxels used (from the bead images) to evaluate the similarity metric during the image registration process. It has to be specified for each scale as follows: dispParam.ffd.evalSampleNum = [coarsestScaleSampleNum,finerScaleSampleNum,finestScaleSampleNum]. Both this parameter and dispParam.ffd.iterNum have to be selected with care if the ASGD optimization method is selected. It is not recommended to use all the pixels/voxels to evaluate the similarity metric if the ASGD method is selected. However, if desired, all samples can be used for the evaluation by setting dispParam.ffd.evalSampleNum = [ ]. For more information on the ASGD method, see the documentation provided with Elastix.

- **dispParam.ffd.beadMask**

  This parameter constrains the selection of the pixel/voxels used to evaluate the similarity metric to the locations provided by a binary mask of the beads. This mask is generated by simple thresholding the bead images.

Parameters for PIV-based Displacements:

- **dispParam.piv.blockSize.xy**

  The PIV algorithm will be run within a 3-level multiscale scheme. This parameter specifies the lateral (in-plane) size of the PIV block for each scale. The size of the block has to be provided in pixels. The scales are sorted as follows: dispParam.piv.blockSize.xy=[coarsestScaleSize, finerScaleSize, finestScaleSize].

- **dispParam.piv.blockSize.z**

  The PIV algorithm will be run within a 3-level multiscale scheme. This parameter specifies the normal (out-of-plane) size of the PIV block for each scale. The size of the block has to be provided in pixels. This parameter is only needed if analyzing a 2.5D TFM experiment. The scales are sorted as follows: dispParam.piv.blockSize.z=[coarsestScaleSize, finerScaleSize, finestScaleSize].

- **dispParam.piv.minbeadPixVal**

  Minimum greyscale value that can be considered to be part of a bead. This parameter avoids error when no beads are present within a given block. This can occur when low bead density is used of the TFM experiment or if the block size is selected too small.

- **dispParam.piv.shiftCalcMethod**

  The method that will be used to calculate the displacements between blocks. Currently, the following methods are provided: normalized cross correlation (xcorr), gradient based shift estimator (grad), and iterative gradient based shift estimator (iterGrad). Both gradient based estimators are included in the dipLib / dipIamge library. For more information on how they work, see:
  Pham TQ, Bezuijen M, Van Vliet LJ, Schutte K, Hendriks CLL. Performance of optimal registration estimators. Proc SPIE. 2005;5817: 133–144. doi:10.1117/12.603304.

- **dispParam.piv.minCorrVal**

  If the normalized correlation method is selected to calculate the displacements between blocks, this parameter specifies the minimum value for the correlation peak that is acceptable to trust the calculated displacements. If the correlation peak value is smaller than the provided threshold for a given block, the calculated displacements for that block are discarded and it will be interpolated/inpainted from its neighboring blocks.

Parameters for Traction Recovery:

- **tracParam.young**

  Young's modulus of the substrate. Units in kPa.

- **tracParam.poisson**

  Poisson's ratio of the substrate.

Parameters for Error Control:

- **errorCParam.tikhonov.weight**

  The error control is performed by including Tikhonov L2 regularization. This parameter controls the amount of regularization applied during the traction recovery process. Set errorCParam.tikhonov.weight=[] for automatic estimation of this parameter.

Results

The resulting displacement and traction fields are stored in a .mat file. See the section above on the user parameters. Specifically:

dispField.X → X component of the calculated displacement field
dispField.Y → Y component of the calculated displacement field
dispField.Z → Z component of the calculated displacement field
tracField.X → X component of the recovered traction field
tracField.Y → Y component of the recovered traction field
tracField.Z → Z component of the recovered traction field
