# Supplementary material for: Free Form Deformation–Based Image Registration Improves Accuracy of Traction Force Microscopy
Source: PLoS One. 2015 Dec 7;10(12):e0144184. doi: 10.1371/journal.pone.0144184 (PMC4671587; doi:10.1371/journal.pone.0144184)
Supplement: S1 Table — Evaluation conditions include 20 realizations with loads with magnitudes of 15%, 10% and 5% of the substrate Young’s modulus, aligned with the X and Z Cartesian direction and distributed over a circular area of 10μm, 6μm and 4μm diameter. (DOCX) [file pone.0144184.s015.docx]

**Supporting Table**

**S1 Table.** **Percentage of realizations providing a single stress footprint.**

|  | **FFD** | | | | | | | | | **PIV** | | | | | | | | |
| --- | --- | --- | --- | --- | --- | --- | --- | --- | --- | --- | --- | --- | --- | --- | --- | --- | --- | --- |
|  | **10um** | | | **6um** | | | **4um** | | | **10um** | | | **6um** | | | **4um** | | |
|  | **15%** | **10%** | **5%** | **15%** | **10%** | **5%** | **15%** | **10%** | **5%** | **15%** | **10%** | **5%** | **15%** | **10%** | **5%** | **15%** | **10%** | **5%** |
| **Load along X-axis** | 100 | 100 | 100 | 100 | 100 | 100 | 100 | 100 | 100 | 95 | 100 | 100 | 100 | 100 | 80 | 100 | 80 | 45 |
| **Load along Z-axis** | 100 | 100 | 100 | 100 | 100 | 100 | 100 | 95 | 95 | 100 | 100 | 100 | 100 | 100 | 100 | 100 | 100 | 95 |

Evaluation conditions include 20 realizations with loads with magnitudes of 15%, 10% and 5% of the substrate Young’s modulus, aligned with the X and Z Cartesian direction and distributed over a circular area of 10μm, 6μm and 4μm diameter.
